# Degradable Plastics and Their Composites: A Comprehensive Review of Production, Properties, and Applications

**DOI:** 10.3390/ma19194230

**Published:** 2026-10-05

**Authors:** Patrycja Bazan, Paulina Majewska, Kamil Badura, Stanisław Kuciel

**Affiliations:** 1 Faculty of Material Engineering and Physics, Cracow University of Technology, Aleja Jana Pawła II 37, 31-864 Krakow, Poland; patrycja.bazan@pk.edu.pl; 2 CUT Cracow University of Technology Doctoral School, Faculty of Material Engineering and Physics, Cracow University of Technology, Warszawska 24, 31-155 Krakow, Poland; paulina.majewska@doktorant.pk.edu.pl (P.M.); kamil.badura@doktorant.pk.edu.pl (K.B.); 3 SHM System Sp. z o.o., Sp. kom., Jana Pawła II St. 82A, 30-444 Libertów, Poland

**Keywords:** biodegradable polymers (PLA; PHA), degradation mechanisms, composites with natural/synthetic fibers, processing technologies, biomass and waste feedstocks (circular economy), design for controlled degradation, mechanical and chemical recycling

## Abstract

Degradable plastics constitute a category of polymeric materials that degrade via biological, chemical, photochemical, or physical processes, with environmental outcomes dependent on the degradation endpoint and disposal conditions. This paper examines the scientific principles, degradation mechanisms, classification, and production processes of these materials, with a focus on poly(lactide) (PLA) and polyhydroxyalkanoates (PHAs). It provides a detailed account of both natural and synthetic raw materials employed in production, polymerization technologies, and methods for forming and shaping products. Particular attention is given to composites with natural and synthetic fibers, their mechanical and thermal properties, and techniques for modifying functional properties. Applications in the packaging, medical, and automotive industries are analyzed, emphasizing the significance of biodegradability and compliance with environmental standards. The life cycle of products, recycling and reuse methods, as well as future research directions, including the utilization of novel biomass raw materials, innovative production technologies, and controlled degradation design, are discussed. To realize the environmental benefits of degradable plastics, it is essential to align their service performance with confirmed degradation processes and viable end-of-life management strategies. Consequently, there is a focus on the examination of residual intermediates and particles, disposal behaviors, and the constraints associated with compostability claims.

## 1. Introduction

In recent years, degradable plastics have garnered significant attention because of their ability to decompose under specific environmental conditions. This characteristic offers the potential to reduce persistent waste, provided that an appropriate end-of-life pathway is established. Unlike persistent conventional polymers, which may accumulate in soil and aquatic environments [1,2,3], degradable plastics can undergo chain scission through biological, photochemical, hydrolytic, oxidative, or other chemical processes. The conversion to products such as biomass, CO_2_, CH_4_, or H_2_O should be specifically described as biodegradation or mineralization when biological activity and the relevant environmental conditions are demonstrated [4]. The origin of the feedstock and its degradability are independent descriptors. Materials considered here include PLA, which is commonly biobased when produced from fermentation-derived lactic acid, and microbially biosynthesized polyhydroxyalkanoates (PHAs), which are used in film packaging, thin-layer technologies, and medical materials [5,6]. Section 3.2 provides an analysis of the processing options for PLA [7]. PHA, recognized for its compatibility with additives and fillers as well as its high biocompatibility, is appropriate for use in the biomedical and food industries [5,6]. The synthesis routes, along with the roles of feedstocks and fillers, are detailed in Section 2.1 and Section 3.1. Fillers derived from waste include invasive plant fibers in poly(3-hydroxybutyrate-co-3-hydroxyvalerate) (PHBV) composites [8,9] and lignocellulosic wastes [10,11]. These methods support circular economy strategies by reducing raw material costs while maintaining adequate mechanical properties. Conventional polymer-processing and additive-manufacturing techniques, such as extrusion, injection molding, and material extrusion (MEX), particularly fused filament fabrication (FFF), are applicable. The mechanical performance is influenced by the material and processing route, rather than solely by the chemistry of PLA [12]. For instance, Ahn et al. conducted a comparison between FDM-processed acrylonitrile–butadiene–styrene (ABS) and injection-molded specimens of the same feedstock, revealing processing-dependent anisotropy and reduced tensile strength under the tested printing conditions [13]. It is important to note that ABS is mentioned here solely as an example of non-PLA processing and not as a biodegradable material. The magnitude and direction of a processing effect should not be generalized without considering the formulation, specimen geometry, loading direction, and test conditions. The production of medical devices is a promising application, with cellulose-modified PLA composites being explored for biomedical purposes [14]. Certain technologies enhance the base polymer by incorporating additives that improve hardness or elasticity. For instance, lignin recovered from pulping liquors can serve as a binder to increase the mechanical resistance of PLA [15], while fiber treatments and compatibilization can alter composite toughness [16,17]. Crystallinity significantly affects the hydrolytic degradation and biodegradation of these materials, as amorphous regions decompose more rapidly than crystalline regions [18,19]. A scenario-specific comparison of production and end-of-life costs is necessary, rather than assuming a disposal-cost advantage (Section 7.4) [20,21]. Predominant recycling techniques include mechanical recycling, chemical recycling, and catalyst-based depolymerization, with method selection contingent on additives and the final product structure. The literature suggests that future research should focus on monitoring mixture stability under storage and usage conditions [16], enhancing mechanical properties by selecting appropriate natural-fiber fractions [22,23], achieving more uniform elongation or modulus of elasticity for processed PLA/BPP [24], and developing technologies compatible with conventional molding processes. Collectively, the advancement of degradable plastics integrates materials engineering, economics, and environmental protection, thereby establishing a foundation for more sustainable production practices in the future. Despite the growing body of literature on biodegradable polymer systems, critical gaps necessitate this review. Studies frequently examine PLA and PHA composites separately, lacking a unified comparison of mechanical, thermal, barrier, optical, and rheological properties across polymer families. The comparison highlights the interconnection between processing, morphology, and service performance in relation to the attainable degradation endpoint. Economic factors encompass uncertainties in production costs [20], life-cycle expenses [21], the EU Single-Use Plastics Directive (SUPD) [25], and extended producer responsibility (EPR) [26]. Additionally, photooxidative degradation, despite its significance in packaging and agriculture, remains insufficiently examined. This review synthesizes materials science, processing, environmental performance, and economic viability within the framework of current regulatory standards.

The guiding thesis posits that the environmental advantages of degradable plastics necessitate aligning service performance with confirmed degradation and practical end-of-life management. Section 2, Section 3, Section 4 and Section 5 analyze the constraints imposed by chemistry, processing, and morphology on both service and degradation. Section 6, Section 7 and Section 8 evaluate the implications of these constraints for applications, disposal, and research priorities. This perspective underscores the importance of a managed end-of-life design [27].

This narrative review is based on searches conducted in Google Scholar, Web of Science, and Scopus, focusing on degradable polymers, processing, properties, degradation, applications, and end-of-life management. Approximately 140 publications were considered during the initial search and screening process, with selection criteria based on relevance, methodological description, and suitability for comparison. Detailed search terms and the inclusion/exclusion criteria are provided in Supplementary Material S1.

## 2. Scientific Basis of Degradable Plastics

### 2.1. Definitions and Concepts

Degradable plastics comprise polymers whose macromolecules can undergo chain scission and a reduction in molar mass. When degradation is induced by environmental factors such as air, light, heat, water, or microorganisms, the term “environmentally degradable polymer” is more precise [28,29]. Degradation may occur as surface erosion or as bulk degradation throughout the sample volume. Biodegradable plastics experience biodegradation through biological activity under specified environmental conditions. For poly(lactic acid) (PLA), a widely studied hydrolytically degradable aliphatic polyester, degradation primarily involves the hydrolysis of ester bonds within the polymer chain. The process necessitates the presence of water to produce oligomers and monomers, while subsequent microbial assimilation is contingent upon appropriate biological conditions [4]. Due to the rigidity of the PLA macromolecule and the presence of methyl groups on the side chains, degradation is generally slow at standard ambient temperatures [17]. However, under specified industrial composting conditions, PLA can meet biodegradation criteria within approximately 180 days, contingent on the test method and specimen characteristics [15]. Polyhydroxyalkanoates (PHAs), biosynthesized by microorganisms, are biodegradable polyesters. In practical applications, alterations to these materials can influence degradation times. Consequently, it is essential to determine the effects of composition and morphology for the specific PHA formulation [30]. Biodegradability refers to the capacity to undergo biodegradation through biological activity; depending on aerobic or anaerobic conditions, ultimate products may include CO_2_, CH_4_, H_2_O, inorganic compounds, and biomass [28]. Conceptually, biodegradation involves depolymerization to lower-molar-mass species followed by bioassimilation and, where applicable, mineralization [31,32]. The evaluation of biodegradation necessitates standardized methods for environmental conditions, which include assessing disintegration or mass loss, quantifying CO_2_ evolution as a measure of mineralization, and analyzing molar-mass changes over time [33,34]. Discrepancies between industrial and natural outcomes can be partially ascribed to variations in ambient temperature, humidity, and the composition of microorganisms. The degradation susceptibility is associated with the polymer’s structure; specifically, a lower molar mass facilitates the hydrolysis and enzymatic degradation of polyesters [28,29]. Additionally, increased chain-segment mobility, linked to lateral substituents with specific stereoregularity, contributes to this process. These structural modifications affect both processing and longevity. Degradable composites are composed of a polymer base integrated with additives that enhance functional properties. Polylactic acid (PLA) combined with plant fibers exhibits significant weight loss even without enzymatic activity, whereas plasticizers reduce brittleness by increasing elongation at break and aiding subsequent mechanical degradation [31,35,36]. These materials are applied in food packaging, medical components, and technical structures that necessitate specific biomechanical and thermal characteristics. Future research is directed towards optimizing composite morphology to enhance biodegradation [37,38] and identifying renewable raw materials that comply with industry standards and EN 13432 [39] for compostable packaging [28]. Thus, chain scission, disintegration, and mineralization answer different questions; environmental benefit cannot be inferred from mass loss alone (Section 7.3).

### 2.2. The History of the Development of Biodegradable Materials

The interest in biodegradable materials originated from the evolving strategies of the plastics industry during the latter half of the twentieth century. By the 1980s, efforts focused on developing partially degradable composites using cellulose fibers and thermosetting resins [16], driven by cost reduction and plant waste management. In the following decade, wood flour-reinforced thermoplastic composites offered greater flexibility compared to thermosetting products, while thermoplastics enabled advanced processing and enhanced control over mechanical properties in composite manufacturing and application design. Concurrently, advancements in poly(lactic acid) (PLA) technology were made. PLA, derived from lactic-acid-based feedstocks, became one of the first widely commercialized biobased polyesters with industrial compostability under specific conditions [22]. Although initially limited by high production costs and low thermal resistance, improvements in polymerization and increased availability of plant raw materials facilitated its widespread adoption. PLA is used in food packaging, medical implants, and surgical threads due to its biocompatibility. Another significant development was research on polyhydroxyalkanoates (PHAs), which are metabolic products of bacteria cultured under nutritional stress [40,41]. PHAs can exhibit high stiffness, UV resistance, and low water permeability and are biodegradable in several biologically active environments, although rates depend on composition and conditions [42,43]. Developed forms include poly(3-hydroxybutyrate) (PHB), noted for its high mechanical strength and suitability for modern additive processing [44,45]. Initially applied in biomedicine and specialty packaging, PHAs were later explored for agricultural uses, such as biodegradable protective nets and greenhouse films [46,47]. Their development was influenced by fuel crises, oil shortages, fossil-resource scarcity, and increasing concerns over plastic-waste pollution [17]. Changes in public opinion have led to legislative measures and research investments in green composites. Consequently, biocomposites began to be produced using methods similar to those for fiber-reinforced polymers (FRP), facilitating the introduction of new products [22]. Over time, the range of degradable polymer systems has diversified. Chemically synthesized degradable polymers, such as poly(ε-caprolactone) (PCL), poly(glycolide) (PGA), and polyanhydrides, have been developed alongside biopolymers such as cellulose and starch [1]. Research efforts to create blends and hybrid composite systems that incorporate biodegradable and/or biobased polymers have intensified, aiming to improve mechanical properties and expand market availability. These developments encompass a range of applications, including the automotive industry, where natural fibers are utilized in interior panels [48,49], as well as electronics, disposable packaging, regenerative medicine, and tissue engineering [42]. Variants of poly(lactic acid) (PLA) composites, reinforced with agri-food waste or specialty fibers such as cordenka, are increasingly employed in the automotive sector due to their high performance and low weight [22]. Over the past two decades, advancements in processing technologies, such as self-piercing riveting and laser bonding of thermoplastic composites, have facilitated their integration into existing industrial processes without requiring substantial modifications to production lines [48]. Simultaneously, research into mechanical and chemical recycling is intensifying to extend the life cycle of biodegradable plastics before end-of-life biodegradation. The development of biodegradable materials illustrates the interaction between external pressures and technological innovation. Although the cost of replacing conventional polymers remains a significant challenge, research indicates a gradual market shift towards renewable and biodegradable alternatives. This development history shows increasing technical versatility, not proof that every commercialized formulation has a suitable disposal pathway (Section 7).

### 2.3. Degradation Mechanisms

#### 2.3.1. Biodegradation

Biodegradation of polymers refers to the degradation induced by biological activity, including that of environmental microorganisms such as bacteria, fungi, and archaea. Under appropriate conditions, this process can ultimately produce CO_2_, H_2_O, CH_4_, and biomass. The mechanism involves enzymatic depolymerization of polymer chains, followed by mineralization or bioassimilation of the resulting fragments. In surface erosion, degradation initiates from the outer surface, whereas in bulk degradation, chain scission occurs throughout the polymer mass, a characteristic observed in plastics with high water permeability and low crystallinity [28]. The rate of biodegradation is influenced by chemical properties, including bond type, stereochemistry, and molar mass, as well as physical parameters such as crystallinity and porosity. A lower molar mass accelerates depolymerization, and amorphous regions facilitate enzyme and water access [33]. Poly(lactic acid) (PLA) undergoes hydrolytic degradation through ester-bond cleavage; products with sufficiently low molar mass can subsequently be bioassimilated and mineralized under suitable conditions [16]. The rate of degradation is influenced by environmental conditions, with results from soil and thermophilic composting being non-interchangeable. Numerical comparisons necessitate consistent endpoints and specimen descriptions, as outlined in Section 5.6. Polyhydroxyalkanoates (PHAs) and their copolymers are biodegradable in soil, compost, and marine environments via enzymes produced by both anaerobic and aerobic bacteria [2]. Aerobic mineralization results in the production of CO_2_, while anaerobic biodegradation may also yield CH_4_. However, neither process provides a definitive completion time for PHAs [4]. The effects of fillers must be interpreted in the context of the specific formulation and testing conditions. Feijóo et al. conducted a study comparing PHBV containing 15 wt.% purified cellulose or woodflour, assessing disintegration on 400 µm films and CO_2_ evolution on cryomilled material sieved through a 250 µm mesh at 58 °C [37]. These represent distinct specimen preparations and endpoints, rather than interchangeable measures of film biodegradation. The study linked the woodflour-containing formulation to delayed conversion, but it does not establish a universal effect of filler loading. An increased composite mass loss may encompass filler loss, soluble-product release, and unrecovered fragments, rather than solely matrix mineralization. Therefore, comparisons must consider filler content and treatment, particle size, matrix composition and molar mass, crystallinity, specimen dimensions, temperature, moisture, inoculum, and recovery procedure. Even bulk CO_2_ evolution from a filled material does not inherently distinguish between filler-derived and matrix-derived carbon [37]. The effectiveness of biodegradation is also influenced by environmental conditions. In vermicomposting, characterized by high humidity and moderate mesophilic temperatures, decomposition mechanisms differ from those in thermophilic composting facilities, which are marked by aerobic bacterial activity [28]. Similarly, the marine environment, with its distinct degrader population and lower temperatures, extends the degradation process despite the presence of active polyester chain degraders. Processing methods also affect susceptibility to biodegradation. The morphology prepared and the subsequent hydrolytic history can alter both the molar mass and crystallinity, as evidenced by hydrolysis-pretreated PLA films [50]. Certain natural plasticizers, such as acetyltributyl citrate, may serve a dual function by enhancing the mechanical properties of composites while facilitating degradation through increased polymer matrix mobility [28]. Plastic waste has the potential to create environmental burdens, the extent of which is contingent upon exposure and waste-treatment conditions [51,52]. When subjected to appropriate biological treatment, biodegradable materials can undergo mineralization, allowing the resultant carbon to re-enter biomass via biological carbon cycling. Research on controlled biodegradation is progressing to forecast service life and end-of-life behavior. For compostable packaging, compliance must be evaluated against the specific criteria outlined in standards such as EN 13432 [39]. Experiments involve chemical matrix modifications, such as reducing crystallinity, and selecting bioactive additives that stimulate colonization by microorganisms. Practically, biodegradable plastics are employed for single-use packaging certified for the intended industrial- or home-composting environment, medical components designed for in situ resorption without surgical implant removal, and agricultural materials that reduce secondary soil contamination after the growing season [53,54]. However, the degradation rate must be balanced with product functionality, including mechanical resistance and gas barrier properties during use. Consequently, the relevant design target is verified conversion in the intended environment, rather than the fastest observed fragmentation.

Identified microorganisms offer more precise evidence than a general reference to bacteria. Nakamura et al. successfully isolated PLA-degrading strains of *Amycolatopsis* sp., specifically K104-1 and K104-2, from soil samples. An extracellular depolymerase, purified from strain K104-1, was found to degrade PLA emulsions and thin films, but it did not hydrolyze PHB or PCL in the substrate tests conducted [55]. This specificity indicates that the presence of a PLA degrader cannot be considered evidence of activity against all biodegradable polyesters. While the isolation and enzyme assays confirm biological capability under laboratory conditions, they do not determine the degradation time for a finished product in soil, compost, or seawater.

#### 2.3.2. Chemical Degradation

The chemical degradation of polymeric plastics involves reactions that cleave bonds within the macromolecular structure, thereby altering their mechanical, physical, and optical properties. In poly(lactic acid) (PLA), these reactions primarily involve the hydrolysis of ester bonds, catalyzed by acids or bases and influenced by temperature and humidity [7]. Water penetrates the amorphous regions of PLA more rapidly than the crystalline areas, accelerating chain breakdown in these regions. As degradation progresses, the crystallinity of the remaining matrix increases because voids left by hydrolyzed amorphous fragments facilitate the reorganization of polymer segments [56,57].

Chemical degradation can occur heterogeneously at the surface or homogeneously throughout the polymer, contingent upon the accessibility of reactants to the interior. In the case of unmodified PLA, the cleavage of main-chain ester bonds is predominant, whereas the cleavage of cross-links is pertinent only to appropriately modified networks [7]. Chain scission becomes significant during repeated recycling processes, as exposure to elevated temperatures and oxidizing environments prompts depolymerization through random ester bond cleavage. These alterations lead to a decrease in molar mass and the release of oligomers or monomers, which may subsequently undergo further oxidation or mineralization.

In the mechanical recycling process involving granule extrusion, residual moisture promotes hydrolysis, while heat and oxygen can promote thermal and oxidative chain damage. This results in a gradual reduction in molar mass and the release of low molar mass volatile products [56]. Although a reduced initial molar mass may enhance subsequent biodegradation, excessive degradation adversely affects mechanical properties. Chemical chain scission during processing must be distinguished from biological conversion after disposal. Residual moisture and thermal exposure are relevant variables; a printing method, heater power, or another processing parameters alone does not define a degradation rate. Hydrolytic susceptibility also depends on the polyester: PLA and PCL films responded differently to the pretreatments examined by D’Amario et al. [50]. The degradation rate is influenced by the stereochemistry of polymers. In poly(lactic acid) (PLA), crystallization can be modulated through the copolymerization of various lactide isomers, such as L-, D-, D, L-, or meso- [7]. Slower crystallization leads to an increase in amorphous phases, making the material more susceptible to penetration by water or oxygen during use or storage. Thus, stereochemistry affects susceptibility, but it does not specify when a measurable change will occur without an exposure protocol. When designing PLA-based composites, it is crucial to consider the impact of additives on degradation. Plasticizers or catalytic additives can reduce hydrolysis time, whereas certain mineral or ceramic components may stabilize the macromolecule or limit contact with environmental reactants [58,59]. The same chain scission that facilitates later conversion can impair processing and service performance; these stages therefore require separate acceptance criteria.

To facilitate a comparison with biodegradation, a common endpoint is necessary. Abiotic hydrolysis may precede biological assimilation, often resulting in consecutive rather than independent competing pathways. In the study by D’Amario et al., compression-molded PLA films with a thickness of 184 ± 84 µm underwent hydrolysis in unbuffered water at 95 °C for 5 h, leading to a reduction in viscosity-average molar mass. During subsequent composting at 58 ± 2 °C, the pretreated PLA achieved nearly complete CO_2_-based biodegradation by day 50, whereas untreated PLA reached approximately 75% biodegradation within the same timeframe [50]. The pretreatment, lasting several hours, measures chain damage rather than mineralization and cannot be translated into a universal factor relative to composting. Instead, the matched composting comparison indicates that pretreatment can reduce the initial lag phase under the specified conditions. Any numerical rate comparison must consider temperature, pH, geometry, pretreatment, and the measured endpoint.

#### 2.3.3. Photooxidative Degradation

Photooxidative degradation can alter the service properties of biodegradable polymers when exposed to light and oxygen. The rate of this degradation is influenced by factors such as the irradiation spectrum and dose, temperature, oxygen availability, and the polymer’s formulation. Laboratory studies have investigated the photoaging of PLA in conjunction with subsequent enzymatic hydrolysis or composting, enabling differentiation between these stages and their endpoints [60,61].

**Figure 1:**
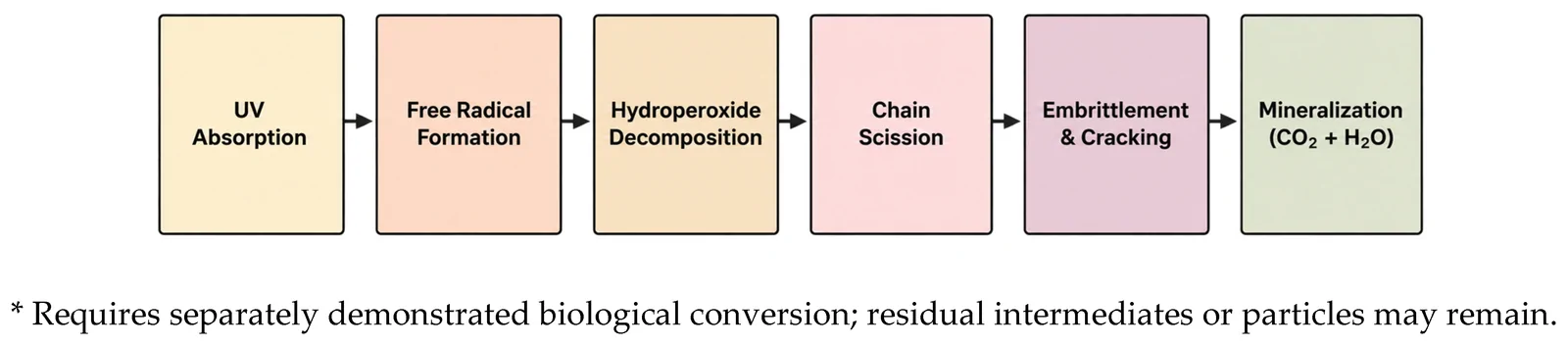
Schematic sequence of photooxidative chain damage and embrittlement. The final arrow to CO_2_ and H_2_O is conditional: subsequent biological conversion must be demonstrated separately and does not follow automatically from photooxidation. Intermediates or polymer particles may remain when conversion is incomplete (Section 7.5) [62,63].

The radical sequence depicted in Figure 1 provides a schematic representation of oxidative chain damage, rather than serving as a universal kinetic model for PLA, PHA, and PBAT. In the case of PLA, the photooxidation mechanism, as discussed by Salač et al., involves the formation of radicals, their reaction with oxygen, the formation of hydroperoxides, and subsequent chain scission [61]. The significant outcome is the loss of chain integrity; however, the formation of radicals or oxygen-containing groups does not indicate the conversion of polymer carbon to CO_2_. Additionally, caution is warranted regarding spectral responses: the PLA study noted a substantial rheological change despite the absence of distinct new FTIR features under the exposure conditions [61].

Photoaging and hydrolysis may interact; however, a change in a single measured property does not constitute a comprehensive biodegradation outcome. Wang et al. documented surface damage and fragmentation of PLA during UV/water aging [62]. These observations indicate material deterioration and potential particle release but do not quantify microbial mineralization. The relationship to biodegradation is more directly demonstrated through paired exposure studies. Salač et al. subjected approximately 100 µm PLA films, with or without an orotic acid nucleating agent, to photoaging in air at 60 °C for up to 100 h using lamps filtered below 300 nm. In subsequent composting experiments at 58 °C, irradiated samples exhibited shorter modeled initial lag phases, while all tested samples nearly achieved full mineralization within approximately 90 days, with similar maximum CO_2_ production rates [61]. Thus, photoaging influenced the initial state prior to composting rather than demonstrating that photooxidation itself accelerated material mineralization. These accelerated exposures should not be interpreted as indicative of universal outdoor lifetimes.

In a complementary experiment conducted by Brown et al., 300 nm irradiation was applied, followed by treatment with proteinase K at 37 °C and pH 7.5. The study found that increased photoaging facilitated ester-bond hydrolysis during the 8 h enzyme assay, even in samples exhibiting heightened crystallinity [60]. The aged polymer was dissolved and recast for the fluorogenic assay, which restricts the transfer to intact products. It is important to note that this experiment measured enzymatic hydrolysis rather than CO_2_ mineralization. Furthermore, samples containing additives in the same study underscore the necessity of evaluating the entire formulation, as opposed to deducing biodegradability solely from the unmodified polymer [60]. The hydrolytic and thermal pathways outlined in Section 2.3.2 may occur alongside photooxidation, with their relative significance contingent upon the exposure conditions.

Consequently, the rate of degradation is contingent upon whether the endpoint is chain scission, mechanical brittle deterioration fragment release, enzymatic hydrolysis, or mineralization. A valid comparison necessitates specifying an identical endpoint and exposure conditions, or it should explicitly outline the successive pretreatment and biological conversion stages [60,61]. The referenced results indicate an interaction between photochemical damage and subsequent degradation, rather than providing a general ranking of photooxidation and biodegradation. The loss of integrity induced by UV radiation remains a variable affecting service life, rather than serving as evidence that released fragments will undergo mineralization (Section 7.5).

### 2.4. Classification of Degradable Plastics

#### 2.4.1. Natural Polymers

Natural polymers, derived from renewable plant and animal biomass, possess structures formed through biosynthetic processes, which impart unique properties distinct from synthetic polymers. Chemically, these polymers include polysaccharides (e.g., cellulose, starch), polypeptides (e.g., silk, wool), and polymers based on lignin or pectins [16,46]. Cellulose, the most abundant biopolymer, forms elongated fibrils within plant cell walls, providing essential rigidity and tensile strength, crucial for biological and composite technologies [64,65]. Lignin, a complex aromatic polymer, binds plant tissues and offers resistance to microorganisms and environmental factors. Extraction methods vary by source; for cellulose, plant fibers are mechanically or chemically separated from lignin and hemicellulose residues, typically through alkaline digestion or sulfite processes. Starch, extracted from tubers or grains, is dried and occasionally enzymatically modified to enhance processability [15]. Proteins such as silk are isolated from natural cocoons using mild chemicals for spinning or film formation. Lignocellulosic fibers reinforce PLA or PHA composites, offering low density and high strength [66,67]. These composites are applied in furniture, eco-friendly construction, structural elements, and device housings. Starch serves as a biodegradable PLA filler, whose cost-effectiveness and availability make it suitable for mass packaging, particularly where rapid compostability is desired [15]. Bamboo fibers are utilized in lightweight structural panels and low-weight packaging materials [66]. Natural plant fibers offer lower production costs compared to synthetic fibers, along with low density, satisfactory stiffness, and potential resource savings that require life-cycle verification [64]. These fibers are renewable, as raw materials can be cyclically sourced from crops or forestry. Many sources are industrial or agricultural waste, such as rice husks or sawdust, thereby supporting the circular economy [53]. However, composites derived from natural polymers have limitations. Microscopic analyses indicate that high concentrations of cellulose fibers may reduce tensile strength and elongation at break due to nonuniform dispersion within the polymer matrix [68]. Enhancing interfacial adhesion and promoting uniform stress distribution can be achieved through improved mixing and appropriate fiber surface modifications, such as silanization or alkalization. Current research focuses on technologies that compatibilize natural polymers with biodegradable polyester matrices, such as PLA or PHBV, using plasticizing or reactive copolymer admixtures. This approach regulates composite crystallinity and adjusts degradation rates to meet specific application requirements. Hybrid systems that blend plant fibers, such as bamboo and linen, are being explored to achieve a balance between hardness and flexibility [67]. This strategy has the potential to expand applications and decrease material costs by using available resources. Natural origin alone does not confer an inherent environmental advantage or a consistent degradation rate for a composite, as discussed in Section 2.1 and Section 7.1. While lignin is one of the more slowly degrading components [53], its incorporation improves mechanical strength, thus extending the lifespan prior to final disposal. Conversely, cellulose demonstrates rapid biodegradability in temperate soils. Advances in techniques, such as multi-phase extrusion and compression molding for mixtures with high fiber content, are rendering natural-polymer products increasingly competitive with traditional plastics [69,70]. The integration of biodegradable polymer matrices with mineral components facilitates applications that necessitate thermal resistance or dimensional stability. Research on natural polymers is advancing towards solutions that combine structural properties with the functional benefits of modern industrially processed biodegradable polymers [16]. The objective remains to develop composites entirely based on renewable raw materials, with mechanical properties comparable to petrochemical plastics and a predictable degradation timeline in accordance with EN 13432 [39] environmental standards. The end-of-life claim must consequently cover the entire formulation, not only the renewable reinforcement.

#### 2.4.2. Synthetic Polymers

Synthetic degradable plastics are chemically synthesized from renewable or partially petrochemical monomers, which are polymerized to control stereochemistry and crystalline structure. Examples include poly(lactic acid) (PLA), polycaprolactone (PCL), poly(glycolide) (PGA), and PLA-PGA copolymers (PLGA) [30]. Unlike the natural polymers discussed in Section 2.4.1, these polymers are entirely human-designed, allowing for the tailoring of material properties through the selection of monomers, catalysts, and reaction conditions. PLA synthesis and stereochemical control are detailed in Section 3.2.1 [7]. The ratio of L-/D- isomers influences crystallization and susceptibility to chemical and biodegradation. Low crystallinity enhances water access and accelerates ester-bond hydrolysis [7]. While PLA exhibits mechanical properties comparable to poly(3-hydroxybutyrate), its thermal strength and gas barrier properties may be inferior to PHB. Consequently, fibers, mineral fillers, or other polymers are frequently incorporated to enhance performance. PCL is a synthetic semicrystalline polyester characterized by high elasticity and a low melting point. Although non-toxic and tissue-compatible, it degrades more slowly than PLA due to its resistance to hydrolysis [30]. To balance service life and biodegradability, PCL composites are often combined with faster-degrading polymers. PGA, with its high crystallinity, offers strong mechanical properties and a shorter degradation period, as crystallinity affects hydrolysis and depolymerization kinetics.

Synthetic degradable polymers are processed using various industrial techniques. Extruding PLA granules results in semi-finished products suitable for injection or compression molding [71,72]. CO_2_ foaming technology facilitates the production of PLA-algal biomass sheets or foams with modified density; the biomass can also provide nitrogen for microbial growth during biodegradation [15]. In the medical field, solvent-forming techniques are employed to produce PLA or PLGA membranes or tissue scaffolds with controlled porosity and resorption time [7].

Research on synthetic degradable polymer composites holds considerable importance. PHB/PCL blends are characterized by immiscibility, evidenced by a decrease in PHB crystallization nuclei density and fractionated PCL crystallization. Conversely, modified PHB/mPCL exhibits partial interfacial compatibility. Additives such as TEC or DCP improve the mechanical properties, including hardness and Young’s modulus, of PHBV without affecting its biodegradability in compost environments containing Pseudomonas mendocina DS04-T [1]. Functional additives significantly impact degradable polymers; for example, lignin or natural fibers enhance PLA stiffness and barrier resistance while altering degradation morphology by hindering enzyme access to amorphous matrix segments [73,74]. Controlling thermal parameters during processing is crucial, as hydrolysis, which is catalyzed by the increasing presence of carboxyl groups (-COOH), can self-accelerate, resulting in the production of lower-molar-mass species. These species undergo distinct biological processes of bioassimilation and mineralization [7]. These materials find applications in compostable packaging, resorbable medical components, agricultural films, and electronic components that require a specific use period before biodegradation. A primary design challenge involves balancing performance with the degradation scenario, as rapid hydrolysis can render a product unsuitable for industrial application. Current research focuses on modifying composite compositions to achieve long-term stability and degradation rates that meet standards [71]. Future research directions include modifications in PLA stereochemistry, hybrids with biocomponents that promote degradation, multiphase PHA/PCL or PLA/PHA systems for uniform mechanical properties, and investigations into the effects of conductive nanofillers on barrier properties and recyclability [73]. Synthetic biodegradable polymers present a design advantage due to the ability to control synthesis steps, allowing for the customization of mechanical properties, physicochemical barriers, and environmental responsiveness for specific applications. Consequently, the synthetic origin is not a limiting factor; rather, the demonstrable performance and conversion of the chosen formulation are critical.

## 3. The Process of Obtaining Degradable Plastics

### 3.1. Raw Materials for Production

#### 3.1.1. Natural Raw Materials

Natural feedstocks employed in the production of degradable plastics and their composites originate from plant, animal, and microbial sources. In industrial applications, lignocellulosic components, starch, natural proteins, and modified polysaccharides are predominant. These materials are cost-effective and frequently sourced as by-products from other industries, thereby supporting their integration into circular economy strategies [53]. Lignocellulose fibers, comprising cellulose, hemicellulose, and lignin, exhibit varying susceptibilities to degradation, which affects the decomposition rate of the composite. The degradation mechanisms of lignocellulosic components are examined in Section 2.3.1, and their effects should be assessed within the context of the entire composite. Plant fibers are extracted through mechanical methods (e.g., shredding, particle separation) or chemical methods (e.g., alkalization, silanization), which improve adhesion to the polymer matrix. These modifications reduce interfacial tension and increase the homogeneity of PLA or PHBV mixtures. For instance, mercerization of flax or alkalization of rice husk enhances compatibility and tensile strength; however, not all natural fillers improve mechanical performance, as elongation at break decreases with increased unmodified fiber content [75,76]. Starch, primarily sourced from corn, potatoes, or tapioca, serves as a raw material for biodegradable composites and can be used in its unmodified or chemically modified form. Its advantages include complete biodegradability, abundance, and cost-effectiveness, though its high hydrophilicity and low tensile strength under humid conditions are limitations, partially mitigated by compatibilizers or plasticizers [1]. PLA-starch mixtures exhibit high permeability to water vapor and oxygen, which is advantageous for food packaging requiring controlled atmospheres, but unsuitable for applications necessitating gas-tight barriers. Natural materials also include proteins such as silk or keratin and polysaccharides like chitosan or pectin. Chitosan offers antibacterial properties and enhances adhesion between hydrophilic phases and the polyester matrix through amino groups reacting with carboxyl segments [77,78,79]. Pectins function as plasticizers, mitigating material brittleness while preserving the rate of biodegradation. In silk, attributes such as high mechanical strength, low specific gravity, and tissue compatibility are highly valued in biomedical applications. Current research emphasizes the utilization of agricultural and food waste, including nut shells and agave, as economical sources of fiber or lignocellulosic powder. Ground pistachio or almond shells, processed to granulate size, facilitate the formation of PHBV composites with improved soil degradation rates [75]. Particle size plays a critical role in material performance; larger particles may lead to mechanical defects, while smaller particles can weaken interfacial adhesion due to agglomeration. Organic raw materials function as active fillers; for example, incorporating algal biomass into PLA decreases the density of sheets or films via the CO_2_ foaming effect and promotes microbial colonization due to the presence of minerals and nitrogen [15]. These approaches require careful regulation of bioactive additive proportions to avoid excessive porosity, which could undermine the final product’s performance. Industrial methods such as twin-screw extrusion, injection molding, and lamination are utilized to produce multilayer sheets that integrate a base biodegradable polymer with reinforcing or decorative layers [78]. When employing plant fibers, it is crucial to maintain humidity below levels that could cause detrimental effects during matrix melting, as excess moisture can hydrolyze the base polyester during molding. Natural raw materials are tailored for various applications: cellulose and linen are used in lightweight automotive materials (e.g., door panels), bamboo in hard housings for household appliances where products meet specified biodegradation or compostability criteria, starch in plastic bags suitable for home composting, chitosan in microbiologically active packaging that protects food from spoilage, and pectins in biodegradable films for fresh food wrappings. Banana fibers and agave serve as alternatives to synthetic materials for reinforcing architectural elements designed for managed end-of-life recovery or degradation after their functional lifespan [27,80]. Future research focuses on efficient fiber surface modification to optimize compatibility with PLA/PHA matrices and other biodegradable polymer matrices while maintaining cost-effectiveness [76]. Experimental studies combining natural raw materials, such as bamboo and linen, demonstrate synergistic enhancement of hardness and vibration damping [75], with potential industrial profitability through reduced component costs and the marketing advantages of renewable materials. Waste feedstocks must thus be assessed for variability, pretreatment demand, and retained product performance, not credited solely for their low purchase price (Section 7.4).

#### 3.1.2. Chemically Synthesized Polymer Feedstocks

Chemically synthesized polymer feedstocks for degradable plastics are produced through controlled processes, typically utilizing monomers derived from renewable sources or petrochemical components modified for degradability. A polymer matrix based on these materials exhibits a reproducible structure and adjustable properties through modifications in stereochemistry, molar mass, or copolymer composition [7]. Unlike the materials discussed in Section 3.1.1, their production is regulated at the polymerization stage, facilitating predictable performance. Poly(lactic acid) (PLA) is polymer feedstock produced by ring-opening polymerization of lactide. High molar mass requires high-purity lactide for mechanical and thermo-mechanical properties [7]. The process involves the condensation of lactic acid into oligomers, cyclization into lactide, and polymerization catalyzed by suitable initiators. The stereoregularity of PLA, particularly the ratio of L- and D-isomers, significantly influences crystallinity; a high content of one isomer promotes crystalline phases that resist environmental reactants and extend degradation time. PLA is used in packaging and medicine for biocompatibility, but requires modification to improve thermal and mechanical resistance. Other biodegradable polyesters include PCL and PGA. PCL, synthesized from ε-caprolactone by ring-opening polymerization, has good flexibility but degrades slowly, limiting rapid decomposition. [81]. It is insufficient to attribute PGA degradation solely to high crystallinity; factors such as chemical structure, water accessibility, and specimen morphology also play significant roles in governing hydrolysis [81]. Poly(lactide-co-glycolide) (PLGA) copolymers combine lactide- and glycolide-derived units, allowing degradation time to be regulated by monomer proportions. An increased PGA content enhances hydrolysis because short hydrophilic segments facilitate water access to the matrix [30]. In the field of biomedicine, PLGA is employed as a scaffold for tissue engineering and as a carrier for controlled-release drug delivery. Synthetic raw materials also contribute to the development of functional composites. PLA can be enhanced with natural fibers or nanofillers to improve Young’s modulus or gas-barrier properties [82,83]. Composites such as PHB/PCL and PHBV/iPLA utilize plasticizers or compatibilizers to improve adhesion, which in turn affects degradation rates [81,84]. The production of synthetic raw materials involves techniques such as twin-screw extrusion for PLA composites with reinforcing and barrier additives [82], solvent casting of PLGA membranes with porosity, and the preparation of PLA- or PHA/PLA-based filaments for FFF in 3D printing of technical elements [85,86]. In certain industrial applications, CO_2_ foaming is used to reduce the density of PLA sheets, thereby increasing their susceptibility to microbial colonization due to the resulting porous structure [15]. During synthesis, synthetic raw materials can be modified, including changes in chain length, molar mass control, and side-group modifications that influence hydrophilicity. The addition of a naturally derived plasticizer, such as acetyltributyl citrate, to PLA enhances elasticity, reduces brittleness, maintains structural integrity during use, and facilitates hydrolytic degradation [22]. Future research should aim to design synthetic raw materials that maintain mechanical stability throughout the product’s lifespan and degrade predictably under conditions such as composting temperatures or specific enzymatic degraders [16]. Progress is being made in monitoring the morphology of PLA/PHA composites during storage and use, as crystallization rates and the fractions of biobased or natural fibers can influence these characteristics. Efforts are focused on integrating optical, thermal, or electrical functionalities with biodegradability by incorporating conductive or pigmenting nanoparticles that preserve visual and functional parameters until degradation begins [82]. This methodology facilitates applications that include smart packaging and biodegradable components for consumer electronics. As a result, synthetic raw materials provide design flexibility and adaptability across various industries, while ensuring controlled degradation in accordance with established standards. Consequently, the purity of feedstock and stereochemical control are crucial, as they influence both processing consistency and the subsequent degradation process.

### 3.2. Processing Technologies

#### 3.2.1. Polymerization Methods

The polymerization techniques employed in the synthesis of degradable plastics are influenced by reaction mechanisms, catalysts, substrate purity, and process parameters. In the lactide route, the synthesis of polylactic acid (PLA) involves lactic acid condensation, lactide formation, and subsequent ring-opening polymerization. Three primary methods are employed: direct polycondensation, azeotropic dehydration, and ring-opening polymerization (ROP). Direct polycondensation is a simple method requiring minimal equipment; however, it results in low molar mass due to the difficulty of water removal. Azeotropic dehydration improves water removal through azeotropic distillation, thereby increasing PLA molar mass, although it requires precise control of temperature and pressure [15]. ROP is favored in industrial PLA production as it achieves high molar mass (exceeding 100,000 g/mol) with reduced solvent impact. This method typically proceeds without solvents, using stannous octanoate (Sn(Oct)2) and an initiating alcohol [22]. ROP depends on high-purity lactide monomers; any contamination or moisture can reduce chain-length uniformity and the final mechanical properties [82,85]. Technological advancements by Cargill Inc., such as continuous distillation and reactor enhancements, have facilitated the maintenance of solvent-free conditions [7], thereby increasing PLA production and establishing it as the second most commercially established biobased/biodegradable plastic after starch-based materials. Variants of ROP are also applied to ε-caprolactone for polycaprolactone (PCL) and lactide/glycolide copolymerization for poly(lactide-co-glycolide) (PLGA). The polymerization of ε-caprolactone yields a highly elastic material with slow biodegradation, and adjusting the molar mass allows the degradation time to be aligned with biomedical or technical applications [81]. PLGA is synthesized by copolymerizing lactide and glycolide; the monomer ratio influences crystallinity, hydrophilicity, and ester-bond hydrolysis, with a higher glycolide content accelerating matrix breakdown in aqueous environments. In contrast, the polymerization of polyhydroxyalkanoates (PHAs) differs from PLA as it occurs through microbial fermentation. Microorganisms assimilate sugars or fatty acids and synthesize polyhydroxyalkanoates intracellularly. Specific bacterial strains and culture conditions, including nitrogen or phosphorus limitation, are used to tailor PHA side-chain length and the proportion of monomers such as 3-hydroxybutyrate or 3-hydroxyvalerate [46,47,86]. This methodology facilitates the production of homopolymers or copolymers with specific mechanical properties and biodegradation timelines. Certain techniques for creating degradable composites involve polymerizing the monomer with a filler or additive, such as hydroxyapatite nanoparticles in PLA/PHA, resulting in a two-step degradation process where PHA decomposes initially, followed by PLA [87,88]. This approach regulates degradation rates by leveraging the differential susceptibility of components to enzymatic action by microorganisms. In industrial applications, the polymerization technique affects parameters, cost, and environmental impact. Methods utilizing organic solvents may increase burdens due to VOC emissions, necessitating technologies that mitigate emissions or eliminate solvents [15]. Ensuring processing compatibility is crucial, as targeted multiphase mixtures necessitate the distribution of molar mass across phases. Comparative analyses reveal that ring-opening polymerization (ROP) achieves a balance among quality, cost, and environmental factors. Azeotropic dehydration proves advantageous for purifying inexpensive substrates during reactions. Conversely, direct condensation is suitable for small-scale production of specialized materials where high molar mass is unnecessary. Future research should prioritize the development of catalysts that are less toxic than the tin compounds currently employed in ROP and focus on integrating synthesis with in situ compositization technologies. This integration will affect the chemical structure and morphology of the matrix, which can be enhanced by natural or mineral additives [22]. Polymerization conditions must yield a product that retains function for its intended service life, particularly in biomedical devices, where resorption must coincide with healing processes. Ultimately, the polymerization method determines critical performance characteristics of degradable plastics: matrix crystallinity impacts gas and water barrier properties, molar mass affects Young’s modulus and impact resistance, and stereochemical structure influences susceptibility to hydrolysis [82]. The selection and refinement of these methods are crucial for materials engineers who aim to customize products for the single-use packaging market as well as more demanding sectors, such as medicine or biodegradable electronics. While control during synthesis is necessary, it remains insufficient; the end-of-life performance of the shaped product must also be evaluated.

#### 3.2.2. Molding and Shaping

Shaping influences not only the geometry but also establishes the thermal and deformation history, as well as the distribution of fibers, interfaces, and voids, all of which affect the properties of the final product. However, shaping alone does not ensure a specific degradation time. For instance, in compression-molded flax/PLA, variations in preform architectures led to differing porosities and mechanical responses [89]. Furthermore, Alimuzzaman et al. [90] observed that increasing molding temperature and time reduced mechanical properties in the nonwoven systems they studied. Therefore, the process–property relationship must be defined for the specific formulation and operating window, rather than being attributed solely to the process name.

Extrusion is essential for producing films, tapes, and granules. In PLA processing, a foaming agent such as supercritical CO_2_ can be used. When homogenized with molten polymer, it forms a porous structure following a rapid pressure drop at the die exit. The cells then expand under controlled conditions and stabilize during cooling [91,92]. These methods facilitate the production of PLA foams with reduced density, thermal insulation, or shock absorption, suitable for packaging and sports equipment.

In the evaluation of foamed or laminated products, it is essential to differentiate between total porosity and accessible pore structure, as well as to report on layer thickness and composition. A reduction in density alone is insufficient to determine water accessibility or biodegradation rate. Mechanical and barrier tests are necessary to characterize service function, while mineralization testing must consider the entire shaped formulation rather than just a biodegradable surface layer (refer to Section 5.6 and Section 7.3). This distinction is crucial experimentally; for instance, the PHBV study by Feijóo et al. utilized films for disintegration but employed milled samples for CO_2_-based biodegradation [37].

Additive processes, such as micro-screw extrusion of woodflour/PHA composites, expand the range of achievable shapes [93,94]. However, the effects on properties are contingent upon the deposited architecture and consolidation. The ABS comparison in Section 1 illustrates the necessity of reporting raster direction and the gap between deposited roads when comparing printed and injection-molded parts [13]. This processing example does not establish biodegradability. Similarly, multilayer structures must be assessed for the combined mechanical, barrier, and end-of-life performance of all layers, rather than solely for the degradability of an exposed layer.

It is also important to distinguish between cooling history and subsequent exposure. In the PLA/orotic-acid study, films were cooled slowly after compression molding to promote crystallization [61], whereas the hydrolysis pretreatment study quenched compression-molded films to inhibit recrystallization prior to testing [50]. These differing formulations and histories do not constitute a controlled comparison of cooling rates, but they underscore why initial crystallinity cannot be inferred from the term “compression molded” alone. Initial molar mass, crystallinity, dimensions, and porosity should be characterized prior to service and degradation tests. In biomedical applications, shaping an appropriate geometry does not ensure sterility, biocompatibility, or a clinically suitable resorption time; these remain distinct requirements of the finished device. Therefore, processing should be chosen to maintain function and reproducible morphology, followed by verification of degradation in the intended environment.

## 4. Composites of Degradable Plastics

### 4.1. Types of Composites

#### 4.1.1. Composites with Natural Fibers

Composites incorporating natural fibers represent a well-developed category of degradable materials, characterized by the integration of polymer matrices—such as poly(lactic acid) (PLA), poly(3-hydroxybutyrate-co-3-hydroxyvalerate) (PHBV), or other biodegradable polyesters—with plant-based fillers. The selection of fibers is influenced by the availability of raw materials, mechanical properties, and their impact on composite parameters. For comparative purposes, synthetic reinforcements (e.g., glass and carbon fibers) and plant-based raw materials, such as jute, flax, hemp, kenaf, bamboo, or abaca, are utilized [53,95]. The primary objective is to substitute synthetic reinforcement with biobased and, where feasible, biodegradable alternatives while maintaining performance standards.

The fabrication process of these composites involves preparing a polymer matrix and treating fibers to enhance interfacial compatibility. For PLA or PHBV, the polymer matrix is compounded or shaped through extrusion or injection molding. Fibers are incorporated either by direct mixing in a twin-screw extruder or by pre-formulating fiber-rich granules. Surface treatments of plant fillers—such as alkalization, silanization, or mercerization—are commonly applied to reduce hydrophilicity and increase adhesion to the hydrophobic matrix [78], thereby improving stress distribution, tensile strength, and hardness.

The incorporation of natural fibers results in variable mechanical effects. Research indicates an increase in the Young’s modulus and flexural strength at specific filler concentrations [22]. Jute, recognized for its low cost, high availability, and high impact resistance, facilitates the construction of lighter structures while maintaining rigidity [95,96]. However, excessive fiber content may reduce elongation at break due to the restricted plasticity of the continuous phase.

Plant fillers influence various aspects of material utilization. The increased hydrophilicity resulting from cellulose and hemicellulose enhances moisture penetration, thereby accelerating hydrolysis and microbial colonization. Research on PHBV demonstrates that samples containing 2% by weight of rice fibers degrade more rapidly than a pure matrix, likely due to diminished interfacial adhesion and the presence of capillary microchannels. When wood serves as a filler, a reduction in the tensile strength of PHBV is noted, which is attributed to a less uniform particle distribution [97,98].

These composites find applications across multiple sectors. In the automotive industry, fibers such as linen, hemp, or kenaf are used to reinforce door panels and interior components, contributing to reduced vehicle weight and improved CO_2_ emissions efficiency [53]. In the construction sector, these composites are employed in lightweight structural panels that offer thermal and acoustic insulation. The furniture industry benefits from their appealing surface texture, derived from fiber structures. Biodegradable packaging reinforced with wood or jute fiber provides enhanced mechanical resistance while remaining suitable for industrial composting. In the medical field, tissue scaffolds based on PLA or PHA, combined with cellulose or chitosan, ensure biocompatibility and controlled degradation of implants. Production techniques are tailored to the characteristics of raw materials, allowing for conventional injection molding of short fibers without obstructing feed channels. For longer fiber bundles, modifications in screw geometry and the extrusion feeding system are necessary to prevent coiling or agglomeration. Lamination enables the creation of multilayer structures where outer layers serve aesthetic or gas barrier functions, while the core contains a high proportion of natural enhancers.

Future research should aim to optimize the adhesion between the polymer matrix and natural fillers and to control morphology through chemical or physical fiber modifications. Enhanced compatibility can extend service life and improve biodegradation predictability at the end of life [78]. Hybrid solutions that combine fibers such as hemp with flax or sisal may achieve a balance between static strength and dynamic flexibility. Surface decorative features can be integrated with mechanical functionality by selecting appropriate fiber cuts and bundle arrangements. Utilizing agricultural waste as filler reduces production impacts and supports the circular economy [40]. Incorporating rice husks, bagasse, or banana waste into PLA/PHA composites facilitates local sourcing and reduces transport emissions. Literature reviews highlight the environmental potential of this group of materials: adaptable processing, extensive industrial applications, and the capacity to design degradation timelines through fiber selection and treatment render them valuable for biodegradable materials engineering [95]. Better adhesion may improve service integrity while restricting water access; reinforcement efficiency and matrix mineralization should therefore be assessed together [37].

#### 4.1.2. Composites with Synthetic Fibers

Composites incorporating synthetic fibers, as opposed to those using natural fillers, exhibit superior mechanical properties due to their homogeneous structure and the controlled production of fibers. In the realm of degradable plastics, these composites are utilized when enhanced tensile strength, Young’s modulus, or dimensional stability is required, while still allowing for matrix degradation. Common fibers include short glass and carbon fibers added to PLA or other biodegradable polyesters, as well as continuous fibers produced in roving forms. The fabrication process requires careful selection of fiber-dispersion methods. Powder-based approaches are less effective for fiber-reinforced composites, as achieving a smooth, uniform powder-fiber layer presents challenges [99,100]. Direct processing methods, such as direct writing or extrusion of fiber-embedded polymer paste, are more efficient as they preserve fiber orientation and minimize gas voids. In additive manufacturing, fibers can be introduced alongside the filament or through impregnation; their orientation and volume fraction determine the final stiffness and strength. Studies indicate that carbon fibers (CF) and carbon nanotubes (CNTs) in composites enhance tensile strength and Young’s modulus [101,102]. Biodegradable polyesters exhibit similar reinforcement effects when such fillers are employed; however, the impact strength may diminish due to brittle fracture. The reinforcement with glass fiber and rice residue yields distinct mechanical and thermal responses. Therefore, comparisons necessitate consistent fiber content, processing conditions, and testing methods [71]. This is attributed to high adhesion and the rigidity of glass. Processing methods influence fiber arrangement. Twin screw extrusion ensures phase mixing and minimizes porosity, which affects fatigue life [101]. FFF enables the orientation of continuous fiber bundles along the component’s direction, producing anisotropic properties beneficial for load-bearing elements with a specific load direction. To fully realize the strengthening potential, the surface of synthetic fibers must be prepared. Coatings or chemical modifications alter surface energy and interfacial tension relative to the polymer matrix, enhancing stress transfer under dynamic or static loads [95]. For glass fillers, silane compatibilizers are standard. By reacting siloxane with the glass surface and polyester groups, they increase adhesive bond strength at the phase interface. Composites with short cut fibers (GF chopped, CF chopped) are easy to dispense for extrusion/injection but provide less reinforcement than continuous fibers. Fabric or cut mat composites enhance multidirectional resistance where isotropy is required. Composites with roving or continuous beams maximize strength but are more challenging to integrate into additive processes.

The primary applications of these systems are found in sectors necessitating a biodegradable polymer base with substantial mechanical load-bearing capacity. These sectors encompass lightweight automotive structures, such as thin-walled interior panels, as well as drones, low-weight aircraft structures, and sports equipment exposed to high dynamic loads. By selecting suitable raw materials, it is feasible to develop a material that adheres to compostability standards for the matrix while preserving structural integrity for a specified duration of use. Future research endeavors to balance the durability of mechanical fibrillar components with the planned degradation of the matrix [99]. Hybrid solutions, which integrate biodegradable PLA/PHA matrices with biosoluble glass fibers or carbon reinforcements, are under investigation; however, the reinforcement and polymer matrix possess distinct end-of-life pathways. Furthermore, efforts are concentrated on incorporating chemically functionalized carbon nanotubes (CNTs) as local stress transfer centers to enhance electrical conductivity, thereby enabling sensor versions of these biodegradable composites capable of monitoring their own technical condition. The ultimate success of implementation will depend on production cost and the flexibility of integration within existing thermoplastic production lines [101]. Degradation of a PLA matrix does not establish biodegradability of glass or carbon reinforcement; recovery or safe management of the residual phase remains necessary [71].

### 4.2. Manufacturing Methods

#### 4.2.1. Compression Molding

Compression molding involves the consolidation of a polymer/filler charge or a fiber preform within a heated mold. The process relies on temperature to facilitate matrix flow, while pressure and dwell time determine contact and consolidation. The resulting properties are also influenced by the preform architecture and thermal history. It is important to note that this method does not inherently ensure a homogeneous, void-free structure. Experiments with flax/PLA provide direct examples of how mechanical performance and residual porosity are dependent on processing conditions [89,90]. The sequence of relevance is as follows: processing conditions lead to consolidated morphology, which in turn affects mechanical and transport responses; each step necessitates measurement.

Akonda et al. conducted molding of unidirectional flax/PLA at 185 °C and 2 MPa for 5 min, resulting in 2.1 mm panels. In contrast, random nonwoven preforms were molded under the same temperature and time conditions but at 4 MPa, producing 2.3 mm panels. The reported porosities were approximately 4% and 18%, respectively [89]. This comparison indicates that increased pressure did not eliminate voids in the bulkier nonwoven architecture. Given that architecture, thickness, and final fiber volume fraction also varied, the study does not isolate the effect of pressure or establish a universal optimum. Therefore, a process specification must encompass preform arrangement and final part structure, in addition to temperature and pressure.

Temperature and dwell time present a trade-off rather than a straightforward improvement. Alimuzzaman et al. observed a decline in mechanical properties with increasing molding temperature and time in their nonwoven flax/PLA systems [90]. These findings define a formulation-specific processing window and do not support the assertion that additional heating invariably enhances impregnation or strength. Similarly, increased stiffness should not be interpreted as evidence of a ductile fracture mechanism. Tensile and flexural responses, impact behavior, porosity, and fracture morphology should be assessed independently before attributing reinforcement effects to a specific failure mechanism.

The same manufacturing decisions influence the specimen presented for degradation testing. A dense panel, an open porous component, and a milled sample expose different geometries and interfaces, meaning evidence obtained for one form cannot establish a numerical lifetime for another. The separate film-disintegration and powder-mineralization protocols in the PHBV study exemplify this limitation [37]. Cooling and nucleation should also be documented: the PLA/orotic-acid experiments demonstrated that increased crystallinity did not necessarily impede subsequent composting under the tested conditions [61]. For packaging, structural optimization must be coupled with barrier performance and comprehensive end-of-life evidence; for medical or automotive components, application-specific functional retention remains a distinct acceptance criterion. Compression molding serves as a means of controlling the initial structure, but it does not constitute proof of compostability, resorption, or environmental advantage.

#### 4.2.2. Extrusion

Extrusion of biodegradable composites is a versatile technique for producing both semi-finished products and final components, characterized by high process flexibility and a wide range of applications. Unlike compression molding, this process involves feeding granules or a composite mixture into an extruder cylinder, where the material is plasticized under controlled temperatures by a transport screw and subsequently pressed through a shaped die. For degradable polymers, it is essential to consider thermal parameters due to their low resistance to overheating. For instance, during melt processing, moist PLA is susceptible to hydrolysis, which results in a reduction in molar mass; it should be noted that approximately 200 °C does not serve as a universal onset threshold [7]. It is crucial to maintain humidity below a critical threshold; drying at 80 °C and inerting the feeding atmosphere can limit oxidative degradation of the polymer melt [15]. Twin-screw extrusion is employed to prepare homogeneous biodegradable polymer blends with functional additives, effectively dispersing plant or synthetic fibers and minimizing filler agglomeration [78]. In PLA composites containing lignocellulosic powders, this process enhances particle wetting and mechanical properties, such as Young’s modulus and flexural strength [22]. The incorporation of bioactive algal biomass reduces sheet density through CO_2_ foaming and increases susceptibility to microbial colonization by enriching the matrix with nutrients [15]. Extrusion also facilitates the integration of foaming agents and compatibilizers in a single cycle. Utilizing CO_2_ or N_2_ under supercritical conditions, it creates a uniform porous structure, resulting in a lighter product with improved insulating properties and energy absorption [91]. This method is applied in the production of shock-absorbing packaging and foam components for appliances. When combining extrusion with foaming, it is crucial to regulate pore diameter, as excessive growth may compromise mechanical structure, while overly small pores may fail to provide the desired cushioning effect. A variant of extrusion involves preparing filaments for FFF additive manufacturing. Polymers such as PLA or PHA/PLA blends can be extruded into strands with controlled diameters (e.g., 1.75 mm or 2.85 mm), then cooled and wound for subsequent use [103,104]. Key process parameters include maintaining melt flow stability, avoiding pulsation, and ensuring appropriate viscosity to guarantee defect-free surface finishes in printing, avoiding issues such as insufficient filling or excessive shrinkage. When extruding composite filaments (e.g., PLA with wood fibers), it is essential to ensure tool resistance to increased abrasiveness and to design nozzle geometries that minimize channel clogging. Single-screw extrusion is predominantly used for monolithic films or technical tapes, often employed as packaging with gas barrier properties. The PLA matrix is frequently combined with polyesters such as poly(3-hydroxybutyrate-co-3-hydroxyvalerate) (PHBV) to enhance barrier properties and delay biodegradation [105,106]. Multi-layer lamination enables the development of gradient materials characterized by distinct degradation profiles. The inner layer serves to protect the product during its usage, whereas the outer layers initiate composting at the end of the product’s life cycle. This technique facilitates the production of barrier membranes or tissue scaffolds with controlled porosity, which can be modified with bioactive particles during matrix plasticization. The literature underscores the necessity of optimizing extrusion processes for biodegradable composites. One strategy involves analyzing the impact of processing conditions on the phase morphology of PLA/PHA, as uneven cooling can lead to heterogeneous crystallinity zones that influence biodegradation rates. Reactive extrusion systems are under investigation, wherein catalysts or cross-linking agents are introduced in situ to modify the polymer’s structure during processing. This includes the controlled reduction of molar mass to decrease composting time without compromising mechanical properties during use. A significant trend is the integration of extrusion into the mechanical recycling of biodegradable polymers, where reprocessed PLA waste is combined with raw material and converted into new products without quality degradation, provided that molar mass loss is managed using structural stabilizers [107]. Additionally, it is crucial to examine how mineral and ceramic fillers, such as micrometric ZnO, incorporated during extrusion, affect the degradation rate and antibacterial properties of the final composite. Balancing catalytic action with adequate processability emerges as a critical design parameter [107]. Extrusion is a versatile process utilized across multiple industry sectors, including packaging films and biomedical components, where precise control is essential. It is important to verify any reduction in melt viscosity following extrusion for potential molar-mass loss before attributing it to enhanced processability.

### 4.3. Property Modifications

#### 4.3.1. Mechanical Modifications

Mechanical modifications of biodegradable composites are utilized to optimize their strength, stiffness, and resistance to dynamic loads in accordance with specific application requirements, while ensuring controlled degradation. These modifications involve selecting fillers and altering the polymer matrix morphology through the incorporation of nanoparticles or fibers. A significant modification includes enriching the PLA/PHA matrix with lanthanum oxide or dysprosium. For example, incorporating 40% Dy_2_O_3_ enhances the Young’s modulus by approximately 50%. This material effectively balances strength and flexibility, making it suitable for applications requiring resistance to stress and deformation, such as load-bearing automotive components or flexible electronics housings. Alterations in mechanical properties may also improve thermal stability, thereby broadening the range of operating conditions. In PLA composites characterized by low impact strength [108,109], the addition of mineral nanofillers (e.g., TiO_2_) further influences surface hardness and abrasion resistance [42]. This modification also enhances chemical properties; the high thermal stability allows usage in high-temperature environments without compromising structural integrity prior to biodegradation. The inclusion of natural fibers in the PLA/PHA matrix enhances Young’s modulus and flexural strength by improving stress transfer between phases [78]. Fillers may reduce elongation at break depending on interfacial compatibilization, necessitating alkaline treatments or silanization to improve fibrous-phase adhesion. Hybrid composites that combine two fiber types achieve a balance between stiffness and dynamic flexibility. Similar effects are observed in PHBV systems with TEC or DCP compatibilizers, which enhance hardness and Young’s modulus without compromising biodegradability under composting conditions [1]. This strategy maintains the material’s life cycle while expanding its technical applications. Research focuses on modifying PLA with elastomeric components or plasticizers to mitigate brittleness. Commercial formulations such as BiomaxStrong or Paraloid™ can increase impact strength by several tens of percent at 2–3% by weight without significantly affecting transparency or smoothness [81]. These additives are advantageous for thin films and medical packaging that necessitate resistance to mechanical damage during transport and use. Chemical functionalization, such as the modification of PLA with maleate anhydride, enhances mechanical properties by improving compatibility with composite components [110,111]. This process facilitates the integration of active components, such as luminous or conductive elements, without compromising structural uniformity. The modification method employed is dependent on the target sector. In automotive applications, the focus is on achieving a high Young’s modulus and low weight; thus, glass fibers or hemp roving are incorporated into the PLA/PHA matrix to create lightweight, load-bearing structures [71]. In packaging, the emphasis is on impact strength and crush resistance, where plasticizers and fine-molecule compatibilizers are used to reduce brittleness without significantly delaying biodegradation [81]. Future research will investigate multifunctional nanocomponents for sensory monitoring and solutions that manage degradation post-service life through gradient crystallinity or filler distribution [112,113], while ensuring life-cycle environmental performance and predictably modulating the transition from functional state to environmental degradation stage for products. The assertion regarding the end-of-life of the modified product cannot be substantiated solely through mechanical improvements; it is also necessary to evaluate the remaining additives and reinforcement (see Section 7.5).

#### 4.3.2. Chemical Modifications

Chemical modifications of biodegradable composites are designed to intentionally alter the macromolecular structure of the polymer or auxiliary components to enhance performance while maintaining or regulating susceptibility to environmental degradation. Unlike mechanical modifications, these modifications operate at the chemical chain level through processes such as cross-linking, functional-group substitution, or interfacial linking between the polymer matrix and filler. In poly(lactic acid) (PLA), chain extension targets terminal hydroxyl or carboxyl groups. Chain extenders, including anhydrides, isocyanates, or epoxides, react with free end groups to form branched structures with increased molar mass [91]. This process enhances tensile strength and processed-state viscosity, facilitating forming methods that require stable flow. Three-dimensionally cross-linked PLA can achieve thermal resistance; however, the effect of increased crystallinity on biodegradation should be monitored. Another strategy involves improving compatibility between the polymer phase and lignocellulosic fibers through condensation reactions or hydrogen bonds. In PLA/lignin systems, strong interactions arise from hydroxyl groups reacting with carboxyl segments, and lignin rings interacting with PLA carbonyl groups [110]. The modified system exhibits enhanced stress transfer and reduced delamination risk under dynamic loads. A similar effect is achieved by cellulose esterification, producing cellulose esters that are more hydrophobic than raw cellulose, thereby reducing surface tension at the poly(3-hydroxybutyrate-co-3-hydroxyvalerate) (PHBV) interface and enhancing adhesion [114]. Composite integrity can be improved by modifying fibers with functionalized acrylic acids, such as grafting acrylic acid onto PLA (PLA-g-AA) with a peroxide initiator and treating rice fibers with a coupling compatibilizer [71]. The esterification reaction between the modified matrix and treated fiber forms covalent interfacial bonds, more than doubling tensile strength compared to the unmodified version. This is significant for packaging and components under high loads. In polyhydroxyalkanoates (PHAs), chemical modifications often alter the monomer fraction during biosynthesis or through reactions on the finished polymer. Incorporation of 3-hydroxyvalerate into PHBV lowers the melting point and enhances flexibility through increased chain mobility. However, the low Young’s modulus and thermal resistance require improvement through anisotropic particles or reinforcing fiber surface modification [97]. Alterations in material properties significantly impact degradation processes, as reduced crystallinity enhances the penetration of enzymes and moisture. Cross-linking serves to reduce the migration of plasticizers or low-molecular-weight additives, thereby improving long-term stability. In PHBV composites, the interaction of hardeners with the matrix and lignocellulosic filler groups enhances hardness while decreasing elongation at break [1]. For each formulation, it is essential to demonstrate the retention of biodegradability following modification. Soil mass loss alone is insufficient to establish matrix mineralization, as detailed in Section 5.6. Chemical modifications also affect processing. Increasing the molar mass of PLA through chain extension prolongs its lifespan before thermal depolymerization, while cross-linked segments maintain viscosity and facilitate the formation of workpieces without surface defects. The use of in situ modifiers during twin screw extrusion eliminates a synthesis step, as evidenced by reactive PLA extrusion involving the functional coupling of diisocyanates [91], while also altering rheological and mechanical properties. Controlling barrier properties is crucial. Nanofillers, such as nanocrystalline cellulose (NCC), serve as crystallization nuclei within the polyester matrix, enhancing thermal resistance and dimensional stability [68]. Their hydrophilic nature also increases moisture sorption, promoting subsequent hydrolytic decomposition, making NCC a promising candidate for degradable food packaging. Future research aims to develop catalysts that are less toxic for ring-opening polymerization (ROP) or cross-linking than tin compounds and to optimize reaction sequences to regulate the post-use degradation rate [110]. Hybrid systems that simultaneously functionalize fibers and the matrix, such as cellulose esterification combined with PLA grafting with bioactive molecules, are being investigated to integrate mechanical and barrier properties and enable release during degradation. This approach may result in plastics that function as packaging components, releasing natural preservatives as composting progresses. Consequently, chemical modifications are crucial for balancing product durability and functionality with predictable environmental degradation in accordance with ecological standards [114]. Chemical compatibility serves to maintain functionality; however, it does not replace the necessity of confirming the degradation endpoint of the modified formulation.

## 5. Properties of Degradable Plastics and Their Composites

### 5.1. Mechanical Properties

**Figure 2:**
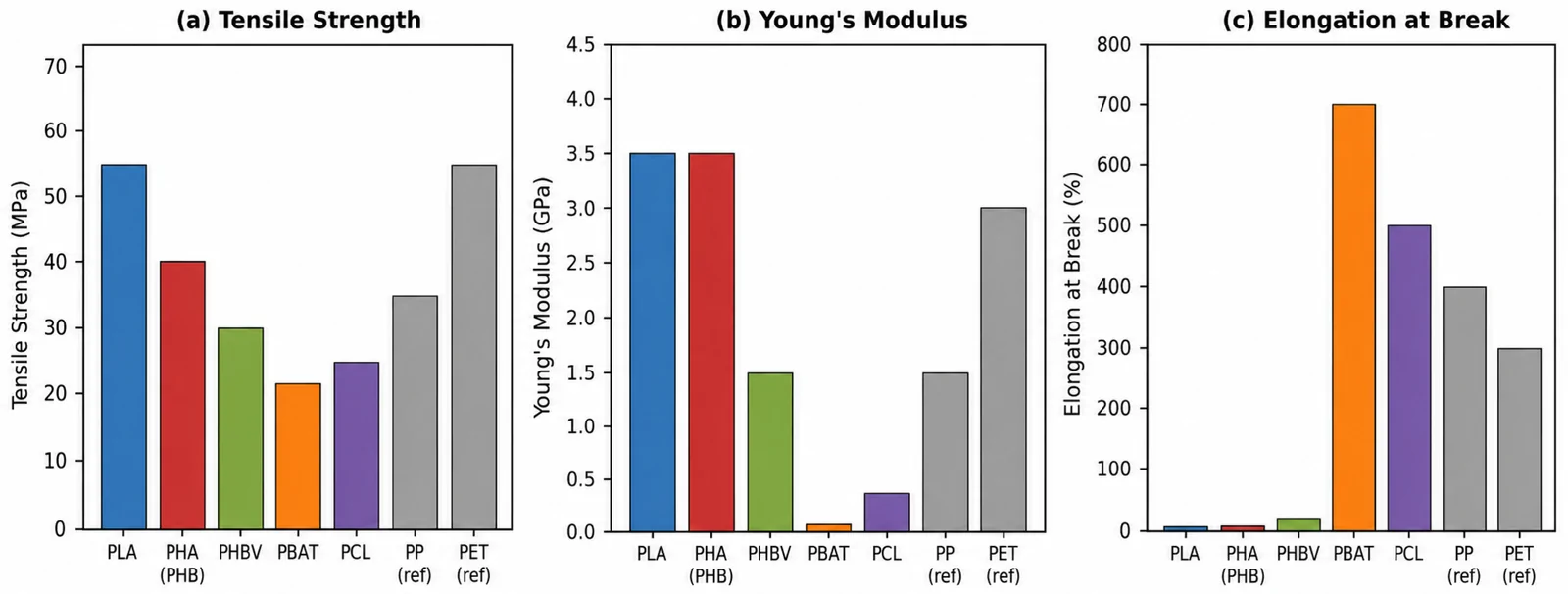
Comparative illustration of tensile strength, Young’s modulus, and elongation at break for the specified polymer families. The axes are linear, and the bars depict single representative values rather than ranges or uncertainty intervals. These values should not be construed as a matched-grade ranking; refer to the qualification in Table 1. [69,81,115].

The mechanical properties of degradable plastics and their composites, including Young’s modulus, tensile strength, and impact strength, are significantly affected by the polymer’s macromolecular structure, filler type, and processing technology. In long-fiber bio-LFT composites, scanning electron microscopy (SEM) demonstrates effective fiber dispersion within the matrix but reveals limited interfacial adhesion, which diminishes elongation and impact resistance compared to compatible systems [69]. Increasing the proportion of PLA in the PLA/PHA matrix enhances performance due to PLA’s rigidity and strength, provided that an acceptable level of flexibility is maintained. In PLA/PHBV-based hybrid composites, the incorporation of 3 phr zinc oxide (HC_3_) improves tensile strength (UTS) and elongation at break (E) compared to pure PLA/PHBV. Despite processing-related degradation of certain components, as indicated by reduced molar mass, mechanical properties are optimized [107]. At higher ZnO content (e.g., HC_5_), degradation becomes predominant, resulting in strength returning to the mixture baseline and a decrease in elongation [62,107]. These findings highlight the necessity for careful selection of mineral additives and control of chemical breakdown kinetics. Poly(lactic acid) exhibits variable mechanical parameters depending on formulation; polymer structure, modifiers, and orientation are critical [81]. PLA is similar to polystyrene in terms of brittleness and low impact strength, while its Young’s modulus and tensile strength are comparable to PET. Its low resistance to plastic deformation under high loads necessitates reinforcement. While glass-fiber reinforcement can enhance the mechanical properties of PLA, establishing its superiority over a specific grade of polypropylene necessitates a direct comparison under equivalent conditions [71]. Biodegradable compatibilizing phases also enhance mechanical properties: PBAT increases impact resistance and allows for a better balance of stiffness and toughness in the PLA matrix [15], while nanocellulose forms a three-dimensional reinforcement system, with CNF fibers increasing Young’s modulus and impact strength while maintaining full biodegradability [115]. In industrial contexts, such solutions are employed to manufacture thin-walled packaging that necessitates enhanced transport resistance. In the field of composite mechanics, modifications to processes are crucial; alterations in extrusion or lamination conditions have a direct impact on the morphology of mixture phases and the transmission of stress. The crystallinity of the matrix and the fiber-polymer interface is regulated, thereby ensuring predictable strength. PHBV serves as an example of the impact of chemical structure, being more flexible than PHB but less impact-resistant than petrochemical polymers. Its susceptibility to modification through mineral admixtures or nanoadditives permits the customization of mechanical properties to satisfy specific market requirements [81]. In practical applications, the mechanical durability of materials must align with the degradation scenario. For instance, automotive materials are required to maintain their load-bearing capacity throughout their service life and subsequently follow a feasible recovery route. In cases where biodegradation is intended, the endpoint must be verified rather than merely inferred from fragmentation. This requires a balance between elastic and strength parameters and the kinetics of hydrolytic degradation or biodegradation. The literature review identifies research directions such as the use of biocompatible nanoadditives, the integration of natural and synthetic fibers to achieve predictable anisotropy, and the development of multiphase PLA/PHA or PLA/PBAT systems to ensure uniform properties throughout the product’s lifespan [69,115]. Thus, the pertinent comparison should focus on the performance maintained throughout the required service life, rather than on the highest isolated strength value. The mechanical properties of the selected polymers are summarized and compared in Figure 2.

**Table 1:** Indicative property ranges compiled for the listed polymer families [1,40,53]. The compilation is not a matched-grade dataset: test temperature, humidity, specimen history, and price basis are not consistently specified. The entries cannot establish universal property, cost, or environmental rankings; biobased content is formulation-specific.

Property	PLA	PHB	PHBV	PBAT	PCL	PP	PET
Tensile Strength (MPa)	50–70	35–45	25–35	18–25	20–30	30–40	50–70
Young’s Modulus (GPa)	3.0–4.0	3.0–4.0	1.0–2.0	0.05–0.1	0.3–0.5	1.0–2.0	2.5–4.0
Elongation at Break (%)	2–10	5–10	15–25	500–800	300–600	200–600	200–400
Tg (degrees C)	55–60	5–10	−5 to 5	−30 to −10	−60	−20	70–80
Tm (degrees C)	150–180	170–180	130–150	110–120	58–65	160–170	250–270
O_2_ Perm. (cc-mil/m^2^-d-atm)	20–50	4–6	10–20	30–50	40–60	300–500	5–15
WVTR × thickness (g-mil/m^2^-day)	25–40	10–20	40–60	100–150	60–100	5–15	10–20
Cost (USD/kg, 2024)	2.0–2.5	4.0–6.0	5.0–7.0	3.0–3.5	4.0–5.0	1.1–1.4	1.2–1.5
Biobased Content (%)	80–100	100	100	0–50	0–100	0	0

### 5.2. Thermal Properties

The thermal properties of degradable plastics and composites are pivotal for their processing and performance under environmental conditions. Key parameters, such as the softening point, melting point, glass transition temperature (Tg), and resistance to thermal degradation, are critical in determining their industrial applicability. Poly(lactic acid) (PLA) is characterized by low thermal resistance due to its limited melting point and low Tg, which restricts its use in high-temperature applications [16]. Nevertheless, PLA remains stable at lower temperatures, rendering it suitable for food packaging in cold environments [22]. Enhancements in thermal resistance can be achieved by incorporating fillers or modifiers. In PLA/PHBV composites, the inclusion of zinc oxide at optimal concentrations improves heat resistance, although excessive amounts may induce degradation during processing [107]. The influence of natural or synthetic fibers on TG/DTA curves is notable; lignocellulosic components lower the initial degradation temperature through the breakdown of hemicellulose and cellulose, whereas glass fibers increase the thermal deformation threshold due to their high-temperature stability [71]. In GF/PLA systems, thermal deformation limits exceed those of plant waste-reinforced PLA (RR). Analysis of DSC data indicates that the presence of mineral particles or nanostructures can modify the glass transition temperature (Tg) and melting point. In the case of PLGA/TiO2 composites, alterations in Tg and decomposition temperature are indicative of thermal behavior; however, these changes alone do not substantiate the heat sterilization of a PLGA/TiO2 device [116,117]. Nanosilicate in PHA elevates the initial degradation temperature and reduces the mass-loss rate during heating through enhanced interfacial adhesion and MMT barrier properties [112]. The storage and conduction of thermal energy are crucial. PHBV blends with spent coffee grounds (SCGs) influence moisture and heat absorption and transport. Peroxides, used as processing additives, enhance heat absorption by forming capillary microchannels. However, high filler contents hinder uniform phase distribution, causing variations in thermal conductivity and potential overheating during operation [118,119]. In molding technology, thermal parameters dictate the process range and component geometry. Tg values of 55–60 °C, as reported by commercial PLA manufacturers, necessitate avoiding direct sunlight and nearby heat sources in applications. Mineral bio-fillers can elevate this threshold, enhancing operational safety. TGA results for metal oxide-infused 3D printed composites indicate that thermal stability varies with the type and amount of additive; samples with low LZFO content achieve higher Tonset values because particles act as physical barriers to macromolecule segment movement [120,121]. Thermal properties are essential in biomedicine, particularly for implants or scaffolds that must endure sterilization without compromising their shape or mechanical integrity. PHBV/wood fiber composites containing hemicellulose demonstrate decreased thermal stability due to the degradation products that expedite polymer degradation during heating [37]. Consequently, the selection of fillers must account for both mechanical reinforcement and temperature-induced chemical reactions. In industrial applications, PLA-CS rotomolding necessitates precise time-temperature management to prevent surpassing the T5% degradation threshold; extended molding below this threshold does not significantly affect performance [80]. Differential scanning calorimetry (DSC) simultaneously verifies the amorphous nature of the poly(lactic acid) (PLA) matrix within these composites. The absence of changes typically associated with crystalline polymers reduces the need for process modifications related to post-cooling crystallization. Future research should focus on integrating enhanced mechanical properties with thermal stability, ensuring that biodegradation or compostability performance is evaluated against pertinent environmental standards. Potential strategies include the copolymerization of monomers with varied crystallization parameters or the alteration of microstructure through additives that act as crystallization nuclei and thermal diffusion barriers. The combination of polyhydroxyalkanoates (PHAs) with decomposing polymers may be utilized to tailor decomposition profiles for applications requiring shape retention under fluctuating temperatures [112], while maintaining a balance between performance and environmental considerations. The decomposition temperature determined by thermogravimetric analysis (TGA) does not qualify as an acceptable service temperature. Separate validation is necessary for dimensional stability, mechanical retention, and any sterilization protocols.

### 5.3. Barrier Properties

Barrier properties are crucial in packaging, as the regulation of oxygen (O_2_), carbon dioxide (CO_2_), and water vapor transmission is critical for determining shelf life and quality. The efficacy of biodegradable polymer barriers is affected by various factors, including their chemical composition, degree of crystallinity, and the incorporation of fillers or nanoparticles. While barrier design is concerned with the functional aspects of packaging [15,33], the assessment of compostability necessitates distinct endpoint testing, as detailed in Section 7.3 [34]. Poly(lactic acid) (PLA) exhibits moderate oxygen barrier properties, with an O_2_ permeability ranging from 20 to 50 cc-mil/m^2^-day-atm, which is superior to polyethylene (PE) but inferior to polyethylene terephthalate (PET). However, PLA demonstrates poor water vapor barrier performance, with a thickness-normalized water vapor transmission value of 30–40 g-mil/m^2^-day (not WVTR alone), limiting its application to dry foods or items with a short shelf life [81]. In contrast, polyhydroxyalkanoates, particularly polyhydroxybutyrate (PHB), offer superior oxygen barrier properties compared to PLA, with O_2_ permeability as low as 4–6 cc-mil/m^2^-day-atm, approaching that of PET and making them suitable for modified atmosphere packaging (MAP) applications [33]. The numerical ranges presented are not directly comparable without considering factors such as temperature, relative humidity, thickness, and method; thus, Table 1 should be interpreted with these limitations in mind. The integration of nanoparticles has been shown to effectively enhance the barrier properties of biodegradable polymers. Specifically, nanoclays like montmorillonite (MMT), when exfoliated and uniformly distributed within the polymer matrix, create a complex diffusion path that significantly reduces gas permeability. The improvements in barrier properties are contingent upon filler dispersion and test conditions; thus, the direction and magnitude of these improvements cannot be determined solely based on clay loading [122]. Similarly, the incorporation of zinc oxide (ZnO) nanoparticles at optimal concentrations of 3 phr in PLA/PHBV blends has been shown to enhance both barrier and antimicrobial properties [107]. Graphene oxide (GO) nanosheets also represent a promising filler for barrier enhancement, with effects that must be related to dispersion, morphology, and the measured transport property [123,124]. It is important to note that barrier enhancement and water access during degradation may compete, necessitating testing on the same formulation.

### 5.4. Optical Properties

The optical properties of biodegradable polymers, including transparency, haze, gloss, and UV transmission, are critical quality parameters for packaging applications. Among these polymers, poly(lactic acid) (PLA) is distinguished by its exceptional optical clarity, with light transmittance exceeding 90% and haze values below 3% for amorphous grades. This positions PLA as comparable to conventional transparent polymers such as polyethylene terephthalate (PET) and polystyrene (PS). Consequently, PLA is favored for transparent food packaging, disposable cups, and display containers within the premium food market segment [81]. The optical properties of PLA are significantly influenced by its crystallinity and processing conditions. Rapid cooling during injection molding or thermoforming fosters an amorphous structure that maximizes transparency, whereas slow cooling or annealing above the glass transition temperature (Tg) induces crystallization, thereby increasing haze and reducing clarity. The incorporation of fillers generally diminishes optical clarity; natural fiber reinforcement introduces light-scattering interfaces, resulting in opaque or translucent composites. However, mineral nanoparticles such as nano-silica or zinc oxide (ZnO), when utilized at low concentrations with sufficiently uniform dispersion, can preserve reasonable transparency while enhancing mechanical and barrier properties [107].

Polyhydroxyalkanoate (PHA)-based polymers, particularly polyhydroxybutyrate (PHB) and poly(3-hydroxybutyrate-co-3-hydroxyvalerate) (PHBV), typically exhibit lower transparency than PLA due to their higher crystallinity and larger spherulite size. The spherulitic structure of PHB, with spherulite diameters often exceeding 50 micrometers, effectively scatters visible light, resulting in a characteristic opaque or milky-white appearance that limits its application in contexts requiring optical clarity [1]. UV stability is another crucial optical consideration. Unmodified PLA transmits a substantial portion of UV-B and UV-A radiation, which can lead to photooxidative degradation of both the packaging material and the enclosed product. The incorporation of UV-absorbing additives, such as benzotriazole derivatives or ZnO nanoparticles, can selectively block UV transmission while maintaining visible light transparency. Recent advancements in biobased UV-absorbers, including lignin-derived chromophores and flavonoid extracts, offer a pathway to fully biobased transparent packaging with enhanced UV protection [68]. Optical clarity or UV shielding pertains to service function rather than biodegradability and should not be used as a substitute for it.

### 5.5. Rheological Properties

The rheological properties of biodegradable polymer melts are essential for evaluating their suitability in various manufacturing processes, including extrusion, injection molding, blow molding, and fused filament fabrication (FFF). Poly(lactic acid) (PLA) exhibits typical shear-thinning behavior, with a zero-shear viscosity ranging from 3000 to 10,000 Pa-s at temperatures between 180 and 200 degrees Celsius. The power-law index for PLA generally ranges from 0.3 to 0.6, indicating moderate shear sensitivity. For FFF, it is essential to consider molar mass in conjunction with temperature, shear history, and formulation, rather than treating it as a universal printability cutoff [7,31].

The rheological characteristics of PLA/PHA blends are predominantly determined by the phase morphology and the interfacial interactions between the blend components. For immiscible PLA/PHBV blends, viscosity follows a logarithmic mixing rule at low shear rates; however, deviations occur at higher shear rates due to droplet deformation. PBAT exhibits significantly lower melt viscosity compared to PLA or PHA, making it a valuable component for enhancing processability [91]. The viscoelastic properties, specifically the storage modulus (G′) and loss modulus (G″), are essential for foaming applications. The melt must possess sufficient elastic strength to stabilize gas bubbles during expansion. Rheology thus connects processing history to functionality. However, a reduction in viscosity does not indicate a biological conversion of the polymer.

### 5.6. Biodegradation Rates Under Specified Environmental Conditions

The rate of biodegradation is highly dependent on specific conditions and is affected by polymer characteristics, environmental factors, and the geometry of the specimen. Compostability standards, such as EN 13432 [39] and ASTM D6400 [125], outline biodegradation criteria (typically ≥90% within ≤180 days) along with distinct requirements for disintegration and compost quality/ecotoxicity. A temperature of approximately 58 ± 2 °C is frequently employed in laboratory composting; however, it does not universally apply to all biodegradation processes. These criteria serve as acceptance benchmarks rather than universal degradation timelines for all PLA or PHA products [39,125]. Environment-specific experiments demonstrate the limitations of a universal ranking. Brdlík et al. assessed CO_2_ evolution for PHBV formulations under thermophilic composting conditions, whereas their comparisons in freshwater and vermicomposting utilized mass loss as a metric; these endpoints are not suitable for consolidation into a single rate [28]. Feijóo et al. conducted a study on PHBV containing 15 wt.% cellulose or wood flour at 58 °C. They employed 400 µm films for disintegration experiments and utilized cryomilled material, which was sieved through a 250 µm mesh, for CO_2_-based biodegradation analysis [37]. It is crucial to note that these results should not be interpreted as mineralization measurements of the intact films. Experiments conducted in various environments have demonstrated that blending PLA with PCL can alter the home-composting performance when compared to pure PLA [126]. Therefore, a comprehensive comparison necessitates consideration of formulation, thickness, environment, duration, and endpoint, rather than relying on a generalized “years to disappear” metric for a polymer family.

## 6. Industrial Applications

### 6.1. Packaging Industry

The packaging industry constitutes a vital sector for biodegradable and compostable plastics and composites, driven by regulatory mandates to reduce plastic waste and the demand for materials that adhere to compostable packaging standards. Polymers such as PLA, PHA (including PHBV), and blends with additives are predominantly employed [15,81]. Production depends on the raw material structure and the intended application. PLA grades formulated for the intended food-contact application are processed into films, bottles, cups, and trays through injection molding, thermoforming, or sheet extrusion]. PHBV, whether pure or reinforced with natural fibers, serves as an alternative to PP or PS in lightweight packaging requiring high rigidity and a sustainable life cycle [33]. Additives like acetyltributyl citrate (ATBC) act as plasticizers, promoting degradation by enhancing polymer-chain mobility [15]. Packaging materials include biobased and/or biodegradable polymers such as PLA, thermoplastic starch, and PHAs, as well as biodegradable polyester blends. PLA is prized for its transparency and ease of coloring, making it suitable for premium food packaging [81]. PHAs provide robust gas barriers for active packaging that maintains products in controlled atmospheres [33]. Polymer blending is utilized to optimize mechanical properties. PLA/PHBV blends at ratios of 75/25 or 50/50 reduce PLA brittleness and improve gas permeability; PBS-containing variants achieve a balance of Young’s modulus, tensile strength, and elongation at break for environmentally friendly packaging. Active packaging formulations necessitate evidence of product-specific preservation and migration, as indicated by reference [84]. Manufacturing techniques are customized based on the number of layers and barrier requirements. Single- or multi-layer extrusion processes yield monolithic films or laminates, such as PLA/PHBV or PLA/PBS, with parameters tailored for foods stored under different humidity conditions. The literature on PLA packaging provides a discussion on active food-packaging applications. In industrial contexts, the relationship between properties and post-use degradation of such packaging is closely scrutinized. For compostable systems, the objective is to meet specified biodegradation, disintegration, and compost-quality criteria within the time limits of the relevant standard; accelerated degradation may be beneficial for disposable fast-food dishes packaged in PLA/PHBV films with high elongation at break due to added plasticizers [84]. Conversely, variants with increased crystallinity, such as PHB enriched with pine radiata fiber, are engineered as reusable containers with an extended service life prior to the onset of degradation [34]. Future research underscores the need to enhance barriers to water vapor and oxygen while ensuring compliance with compostability standards under EN 13432 [39]. Hybrid solutions that incorporate the biodegradable phase with thin mineral nanolayers, such as SiO_2_ or ZnO, are being evaluated to improve the mechanical resistance and antibacterial properties of the barrier layer [62]. A developing trend involves the use of agricultural waste as a cost-effective source for biodegradable polymer blends; for example, plant-derived fibers are investigated as fillers in PLA biocomposites [36]. The integration of biodegradable polymer composites in packaging is increasingly linked with 3D printing for the production of small batches and prototype molds, which are sealed using a PLA/PBS foil sheet. This method offers benefits such as the elimination of expensive industrial molds and the rapid adaptation of geometry to align with the manufacturer’s product range, while compostability must be assessed independently of the forming technique (Section 7.3). The packaging industry is transitioning from basic starch–PLA films to advanced laminates that combine mechanical properties with preservation or controlled atmospheres. This shift addresses environmental and legislative demands and supports the circular economy [15,33]. The environmental argument is contingent upon the preservation of food protection and the availability of a disposal route for the entire package, rather than solely focusing on the polymer label [27].

### 6.2. Medical Industry

In medical applications, degradable and bioresorbable polymeric materials are increasingly employed in devices, implants, and therapeutic systems that necessitate a controlled functional lifespan followed by degradation and, where demonstrated, bioresorption. Unlike the packaging materials discussed in Section 6.1, which prioritize rapid compostability post-use, medical materials require degradation kinetics that align with healing or resorption processes. Research on PLA-based materials focuses on their potential applications in the biomedical field [81]. Concurrently, studies on PHBV are concerned with scaffold fabrication and drug delivery systems [30,127]. It is essential to validate the biocompatibility, sterilization tolerance, and degradation of the final device. Components based on PLA can be manufactured through solvent-forming for tissue scaffolds, FFF 3D printing for custom-fitted implants, and injection molding for precision surgical components [82,83]. In structures that contact tissue, PLA must maintain porosity to facilitate cell infiltration and metabolite transport; bioactive ceramics such as β-tricalcium phosphate (β-TCP) and hydroxyapatite provide reinforcement and support osseointegration [81]. The PLA matrix degrades within the required timeframe, while ceramic particles persist as a mineral scaffold for bone reconstruction. PHAs, produced via microbial biosynthesis, have metabolites that are tolerated by the body, making them suitable for gastroenterological implants or stents [30]. Monomer composition can affect degradation; however, the resorption period is specific to both the device and the site [30]. PHBV matrices have been studied for their potential in drug encapsulation and controlled release [127]. Experimental nanocellulose-enriched PLA/PHA scaffolds enhance the Young’s modulus and impact strength while maintaining biocompatibility [81,84]. Although 3D printing facilitates the creation of implants, maintaining mechanical strength during therapy necessitates precise control of printing parameters and internal structure [82,83]. Current bioprinting research emphasizes optimizing scaffold pore size and the uniform distribution of bioactive additives, as these factors directly influence osteoblastic colonization and angiogenesis. In industrial applications, polymer compatibility with implant surfaces is also crucial. Chitosan admixtures impart antibacterial properties to scaffolds and facilitate tissue integration through interactions between amine groups and collagen. PLA/hydroxyapatite composites, fabricated via extrusion or compression molding, are used to create bone implants tailored to defect sites. The degradation of the polymer matrix exposes the mineral phase, supporting bone reconstruction. Controlling in vivo degradation is crucial for implants, which must maintain mechanical integrity throughout tissue regeneration and subsequently degrade without releasing harmful byproducts. Rapid degradation can undermine stability before healing is complete, while slow degradation may require surgical removal. Consequently, the selection of composition and morphology should be based on degradation evidence specific to the device and site, rather than on a presumed universal enzymatic pathway. Applications extend beyond orthopedics and dentistry to include barrier membranes for reconstructive surgery and resorbable sutures. In the latter, tensile retention time is critical; PGA-based threads degrade more rapidly than PLA/PLGA, making them more suitable for superficial wound healing [30,128]. Recent studies highlight the integration of conductive nanofillers to monitor implant conditions or trigger drug release via electrical stimuli. Additionally, gradient crystallinity scaffolds with varying degradation rates and multi-material printing techniques are utilized to align resorption schedules with treatment phases [82,83]. In medical applications, plastics must adhere to stringent chemical and biological purity standards. The biosynthesis of polyhydroxyalkanoates (PHAs) and the polymerization of lactide are optimized to eliminate toxic catalyst residues, such as tin in the ring-opening polymerization (ROP) of poly(lactic acid) (PLA), and to ensure the absence of microbial endotoxins. These applications include bioresorbable tracheal scaffolds and temporary vascular prostheses. Advances in processing and modification enhance the mechanical and degradation properties of materials for surgical procedures or pharmacological therapies [30,81]. In this context, hydrolysis refers to bond cleavage, while bioresorption necessitates not only the biological removal of the material and its products but also the clearance of these substances. A mere reduction in strength or mass does not suffice to confirm clearance.

### 6.3. Automotive

In the automotive industry, the use of degradable plastics for interior components and technical systems with predetermined service lifespans is increasing. This trend is driven by the need to reduce vehicle weight, fuel consumption, and greenhouse gas emissions, alongside a focus on recycling in line with circular economy principles. The primary challenge is to ensure mechanical durability during use and to manage end-of-life recovery or degradation after the vehicle’s service life. Automotive composites based on degradable polymer matrices can be produced using twin-screw extrusion, compression molding, injection molding, and additive manufacturing for prototyping. Poly(lactic acid) (PLA), poly(3-hydroxybutyrate-co-3-hydroxyvalerate) (PHBV), and blends with plant fibers are often formed into flat panels by compressing heated granules (160–180 °C), followed by cutting and assembly [22]. The extrusion of technical profiles facilitates the creation of structural elements for door panels, storage compartments, or dashboards [53]. It is essential to maintain raw material moisture below the hydrolysis threshold in PLA prior to forming, which is achieved through drying and inerting [7]. Natural-fiber composites offer weight reduction while maintaining adequate stiffness [78]. The selection of composites involves balancing component strength and weight, with the high Young’s modulus of natural fibers being advantageous. PLA/kenaf composites achieve a Young’s modulus of almost 8 GPa and a tensile strength over 100 MPa [22], making them suitable for load-bearing interior elements. The incorporation of coconut or jute fibers in polyhydroxyalkanoates (PHAs) enhances impact resistance and reduces brittleness, which is crucial for passive safety. The integration of continuous glass fibers during FFF 3D printing or lamination results in mechanical performance comparable to traditional fiber-reinforced plastics (FRPs) [82]. GF/PLA composites offer mechanical reinforcement; however, a valid comparison with polypropylene necessitates equivalent composition, geometry, and testing conditions [71]. Performance can be customized through mechanical and chemical modification of the polymer matrix. Natural plasticizers such as ATBC improve flexibility without compromising compatibility [15], while acrylic acid grafted onto PLA enhances adhesion to lignocellulosic fillers and strengthens the phase interface. These treatments are vital in automotive applications, where dynamic loads can result in delamination. Thermal resistance is critical under cabin insolation and fluctuating temperatures. The inclusion of mineral fibers or ceramic additives enhances deformation thresholds, with GF/PLA composites surpassing RR/PLA (Rice Residue) composites at operational temperatures [71]. PLA/PHBV mixtures with zinc oxide improve thermal stability; however, the composition requires optimization to prevent matrix degradation during processing [107]. These materials are suitable for interior components such as door panels, center consoles, trunk covers, and soundproofing elements made from foamed PLA/PHBV. Furthermore, air vents and storage housings are designed with hemp-fiber-reinforced PHA to improve impact resistance. In prototype parts, 3D printing facilitates the production of service tool holders and short-series covers for electrical components, which can subsequently be disassembled with the base matrix in compliance with environmental standards [82]. Research in the automotive industry on degradable plastics focuses on enhancing the thermomechanical resistance of composites while maintaining the intended service life and end-of-life performance. EN 13432 [39] specifically applies to packaging intended for recovery through industrial composting and should not be considered a general automotive degradation standard. This includes developing mechanical recycling methods for PLA- or PHA-based automotive products prior to complete biodegradation and advancing hybrid fiber-matrix systems that allow controlled property loss after a predetermined service life, consistent with vehicle schedules. The integration of conductive nanomaterials into biodegradable composites is also being explored to enable sensor functions for condition monitoring and replacement before degradation compromises driving safety [101]. This market segment has the potential to advance degradable polymer technology by balancing performance with environmental considerations for next-generation vehicles. In this sector, priority is given to durability and recoverability, as programmed fragmentation post-use does not constitute a sufficient end-of-life strategy [27].

## 7. Environmental Impact

### 7.1. Product Life Cycle

The life cycle of a product, particularly degradable plastics, includes all phases from raw material acquisition through production and use, to end-of-life processes such as recovery, degradation, recycling, or disposal. Plastics like poly(lactic acid) (PLA) or poly(3-hydroxybutyrate-co-3-hydroxyvalerate) (PHBV) undergo both physical and chemical transformations throughout this cycle, which can be strategically planned during the material design phase. Unlike petrochemical products with extended shelf lives, degradable plastics necessitate the tailoring of structural parameters to suit the intended end-of-life degradation scenario, ensuring that emissions and disposal residues adhere to environmental standards [28].

The raw material stage significantly influences the product’s carbon footprint. Biodegradable polymers may be derived from plant biomass, such as corn starch for PLA, or synthesized by microorganisms, as seen with polyhydroxyalkanoates (PHAs) and PHBV [37,38]. This selection affects the material’s availability, mechanical properties, and degradation time. The manufacturing process involves polymerization, typically using the lactide ring-opening method for PLA, followed by shaping through extrusion, injection, or lamination [22]. Polymerization parameters can be adjusted to achieve the desired crystallinity or molar mass, impacting mechanical resistance during use and susceptibility to hydrolysis or oxidation.

The operational phase focuses on ensuring that performance standards are maintained throughout the designated period. Packaging or medical products must retain structural integrity, including mechanical resistance and control over gas barrier or moisture permeability [33]. In automotive applications, PLA/PHBV composite components must demonstrate rigidity under varying thermal conditions [71], while PLA/hydroxyapatite bone scaffolds are required to remain stable in vivo until tissue regeneration is complete [30]. Additives such as plasticizers and natural or mineral fibers are used to tailor the functional lifespan.

End-of-life degradation is influenced by microorganisms, enzymes, water, oxygen, light, or temperature, initially producing lower-molar-mass products. Under biodegradation conditions, further mineralization may result in the formation of CO_2_ or CH_4_, H_2_O, inorganic products, and biomass. Biogenic CO_2_ can be reincorporated into biomass via photosynthesis, a process known as carbon cycling, rather than a direct closed-loop recovery of polymer material [22]. PHBV and its composites possess the ability to biodegrade under conditions such as industrial composting, soil, or aquatic environments. However, the rates and extents of this degradation are dependent on both the formulation and the specific environmental conditions [37,38]. According to EN 13432 [39], at least 90% biodegradation must occur within a maximum of 6 months, with disintegration requirements stipulating that, after 12 weeks, no more than 10% of the initial dry mass should remain as fragments larger than 2 mm [28]. A crucial component of closing the cycle involves implementing mechanical or chemical recycling before complete degradation. Mechanical shredding of PLA/PHBV waste followed by re-extrusion prolongs the material’s lifespan with minimal raw material consumption; however, this process requires monitoring of molar mass reduction due to previous exposure to heat and humidity [7]. Chemical recycling through catalytic hydrolysis enables the recovery of monomers such as lactic acid or 3-hydroxy fatty acids for reuse in synthesizing polymers of quality comparable to virgin products. Comparative analyses of greenhouse gas emissions suggest that biobased and/or biodegradable polymers may surpass conventional plastics in specific life-cycle metrics. It is essential for life-cycle assessments to compare equivalent delivered functions while explicitly stating the assumptions regarding energy, treatment, and carbon accounting [21].

### 7.2. Recycling and Reuse

The recycling and reuse of degradable plastics enhance material functionality and decrease reliance on virgin raw materials. These processes encompass mechanical, chemical, and biological methods, with their efficacy contingent upon polymer structure, composite type, and technological conditions. Mechanical recycling involves the shredding of waste, drying, and replasticizing through extrusion or injection. For PLA used in FFF, reprocessing may diminish tensile strength and cause material embrittlement due to molar mass degradation [129,130]. The application of bioinspired coatings, such as polydopamine (PDA) on PLA pellets prior to extrusion, enhances the mechanical properties of the recovered filament, potentially improving reuse performance. In PHBV-based biocomposites modified with buckwheat husk (GBH), successive recycling cycles stabilize the microstructure, mitigating initial mechanical declines through improved composite homogeneity, uniform filler distribution, and enhanced matrix wetting [131]. This process reduces water absorption and decelerates biodegradation during storage. To avert excessive chemical degradation during mechanical recycling, processing parameters must be meticulously controlled. Prolonged exposure to oxygen or moisture at elevated temperatures results in ester bond rupture and molar mass reduction [56], which can be mitigated by inerting the atmosphere and gradually heating dried granules. Chemical recycling involves depolymerization to recover monomers such as lactic acid. For PLA, hydrolysis at elevated temperatures (170–200 °C) can degrade the polymer to oligomers within 90 min. These intermediates can be re-polymerized to achieve properties comparable to virgin material. Furthermore, hybrid methods enhance mechanical recycling with compatibilizers or stabilizers. For PHBV, the incorporation of organic plasticizers (TECs) and cross-linking initiators (DCPs) maintains composite hardness and elasticity after multiple processing cycles [1,2]. The success of these treatments hinges on balancing structural reinforcement with the preservation of biodegradability under the relevant end-of-life specification; for compostable packaging, EN 13432 [39] is applicable. Reusing degradable plastics does not require remolding them into identical products; rather, their components can be adapted for diverse applications. For example, automotive PLA/PHBV components can be dismantled after their service life and repurposed into reusable packaging or technical components with shorter service durations, thus extending the material’s functional phase before environmental degradation occurs. Industrial recycling and reuse strategies must consider sector-specific requirements. In the context of food packaging, ensuring food-contact safety post-recycling is essential; in the medical field, reuse is feasible only for semi-finished products or raw materials that meet purity and biocompatibility standards. In automotive applications, remanufacturing panels or profiles from melted PLA/natural fiber mixtures can reduce waste destined for composting or incineration. Literature analysis indicates several research directions: the development of compatibilizers tailored to biodegradable polymers, the use of Reactive Extrusion with molar-mass regeneration, the creation of composites with controlled property degradation while distinguishing degradation from biodegradation, and the optimization of drying and inerting processes to minimize parameter loss during recycling [129,130,131], thereby facilitating the creation of functional “second life” materials. Material recovery and mineralization represent distinct outcomes: recycling preserves the value of polymers or monomers, while composting necessitates a separately justified function within the waste management system [27].

### 7.3. Standards and Certification

Certification of compostable plastics in accordance with recognized standards is essential for verifying product performance, preventing greenwashing, and supporting defensible environmental claims. Generic biodegradability claims must also specify the environment and relevant time scale. The standards discussed below regulate compostability in specified environments and define distinct criteria for biodegradation, disintegration, composition, and ecotoxicity [28].

Assessing the compostability of polymeric materials necessitates the use of standardized test procedures, as merely labeling a material as “biodegradable” does not specify the environment, time scale, or required extent of biodegradation or mineralization. For plastics intended for industrial composting, the most commonly referenced documents are the European standard EN 13432 [39], the American ASTM D6400 [125], and the international ISO 17088 [132]. These standards primarily pertain to the controlled, aerobic, and thermophilic conditions characteristic of industrial or municipal composting facilities. EN 13432 is primarily applicable to packaging, whereas ASTM D6400 and ISO 17088 have a broader scope, encompassing plastics and products made of plastics. Despite differences in application scope, these standards assess compostability based on similar criteria. These include the composition characteristics of the material, aerobic biodegradation, disintegration during composting, and the impact of the tested material on the quality of the resulting compost. This implies that mere mass loss or disintegration of the sample into small fragments is insufficient evidence of compostability. It is also necessary to demonstrate the mineralization of organic matter, typically by measuring the amount of CO_2_ emitted, and to confirm that the material residues do not cause ecotoxic effects.

EN 13432 [39] represents the fundamental European standard for packaging intended for recovery through composting and biodegradation. This standard evaluates the biodegradability of the material, its disintegration during biological processing, and its impact on both the process and the quality of the resulting compost. Under aerobic conditions, a minimum of 90% biodegradation must be achieved within a period not exceeding six months. In the disintegration study, after 12 weeks, no more than 10% of the initial dry matter of the test material may remain as fragments retained on a sieve with a 2 mm mesh, indicating that at least 90% of the material should disintegrate into fragments smaller than 2 mm. Furthermore, the material must not adversely affect the composting process or the properties of the resulting compost.

ASTM D6400 [125], primarily utilized in the United States, applies to plastics and products intended for aerobic composting in municipal and industrial installations where thermophilic conditions are present. In the newer editions of this standard, the biodegradation criterion requires that at least 90% of the organic carbon of the tested product is converted into CO_2_ within a period of no more than 180 days, relative to the control material or absolute value. Regarding disintegration, after 84 days of composting, no more than 10% of the initial dry matter may remain on a 2 mm mesh sieve. The impact of the material on the quality of the compost is also evaluated, including its phytotoxicity and the content of regulated components.

ISO 17088 [132] is an international standard specifying requirements for plastics and plastic products intended for recovery through organic recycling. This standard encompasses four essential elements: disintegration during composting, final aerobic biodegradation, no negative impact of compost on terrestrial organisms, and control of material components. A biodegradation level of at least 90% is required over a maximum period of 180 days. In terms of disintegration, after 84 days, no more than 10% of the initial dry matter may remain on a 2 mm mesh sieve. ISO 17088:2021 also includes extensive ecotoxicity requirements, such as plant growth and earthworm toxicity tests, as well as control of metals and other elements, per- and polyfluorinated compounds, and other hazardous substances.

Biodegradation studies in accordance with these standardization systems often employ conditions around 58 ± 2 °C, corresponding to the thermophilic phase of industrial composting. However, it is important to note that this temperature is primarily a parameter of standardized laboratory methods such as ISO 14855 [133] or ASTM D5338 [134] and does not imply that the actual material in an industrial installation must maintain a constant temperature of 58 °C for the entire 180-day period.

Table 2 illustrates that the requirements of EN 13432, ASTM D6400, and ISO 17088 are largely analogous with respect to biodegradation time and disintegration criteria. These standards mandate not only the disintegration of materials but also a significant degree of biodegradation or mineralization, ensuring that residues do not adversely affect the compost environment. The primary distinctions among these standards lie in the scope of products they encompass and the specific regulations concerning composition and ecotoxicity control. EN 13432 is specific to packaging, ASTM D6400 pertains to products intended for American municipal and industrial composting systems, and ISO 17088 serves as an international specification for plastics intended for organic recycling. A notable limitation of all three standards is that compliance does not guarantee material biodegradability in all environments. Specifically, adherence to EN 13432, ASTM D6400, or ISO 17088 does not confirm rapid biodegradation in soil, freshwater, marine environments, or backyard composters. ISO 17088 explicitly states that it should not be construed as a specification for plastics entering the environment as waste and does not include composting in small household installations. The evidential implications for intermediates, residual particles, and labeling are detailed in Section 7.5.

### 7.4. Economic Analysis and Cost Considerations

The economic feasibility of biodegradable polymers continues to pose a substantial challenge to their widespread adoption. In the case of polylactic acid (PLA), cost analysis should distinctly consider feedstock supply and pretreatment, fermentation and purification, lactide formation/polymerization, utilities, and capital expenses [20,21]. For polyhydroxyalkanoates (PHA), fermentation productivity and polymer recovery/purification are critical cost constraints [4]. Although scaling can distribute fixed costs over a larger output, factors such as feedstock variability, recovery yield, and plant utilization may counteract this benefit. Consequently, waste biomass is not inherently the most economical feedstock [20]. It is essential to differentiate these process costs from resin selling prices, which are further influenced by grade, region, contract size, and date.

Published models underscore the significance of assumptions. Ioannidou et al. estimated a life-cycle cost of USD 1.42/kg for PLA production compared to USD 1.66/kg for biaxially oriented polypropylene (BOPP) in a sugar-beet-pulp biorefinery scenario, where a pectin-rich co-product is valued at USD 3/kg; this does not represent a universal cradle-to-grave packaging comparison [21]. Zhang et al. modeled seven PLA-recycling scenarios, reporting a 22.87% reduction in production costs for a TiO_2_/SiO_2_-catalyzed route relative to their reference recycling process, which does not reflect an observed market-wide saving [3]. For end-of-life decisions, comparisons should employ equivalent delivered functions and incorporate collection, sorting, transport, rejects, treatment, and recovery credits; these quantities necessitate local data rather than generic composting or landfill fees. Policy also influences cost allocation: the Single-Use Plastics Directive (SUPD) encompasses biobased and biodegradable plastics, thus biodegradability does not provide a general exemption [25]. Extended Producer Responsibility (EPR) fee modulation can consider durability, reusability, recyclability, and hazardous substances, but does not automatically confer a discount for a “biodegradable” label [26]. Therefore, a credible economic advantage necessitates a technically feasible product and an available treatment route, with sensitivity analysis for feedstock/co-product prices, scale, and collection efficiency.

### 7.5. Established, Contested, and Unresolved Evidence

A biodegradability claim must specify the material, the receiving environment, the time frame, and the endpoint. Table 3 differentiates between direct observations and extrapolations; the term “unresolved” indicates gaps not addressed by the evidence reviewed, rather than a complete lack of relevant research. Molecular intermediates and microplastic particles represent distinct residual fractions and necessitate different analytical methods [63,135].

These distinctions lead to a limited conclusion: degradation may decrease persistence under appropriate conditions; however, neither fragmentation nor favorable labeling guarantees a net environmental benefit. The studies referenced do not warrant categorizing every intermediate as hazardous, nor do they justify assuming it is harmless. Validation should integrate polymer-specific mineralization, measurements of residual particles and soluble products, exposure-relevant ecotoxicity, and observed disposal behavior. These priorities are further addressed in Section 8.3 and Section 9.

## 8. Directions for Further Research

### 8.1. New Biomass Raw Materials

The utilization of biomass raw materials in the production of degradable plastics is crucial for cost reduction and the expansion of sustainable materials with properties akin to traditional polymers. As elaborated in Section 7.1, biomass sources encompass conventional lignocellulosic waste, crop fibers, atypical agricultural waste fractions, and microbial resources. The initiative to complete the product life cycle has led to an increased use of raw materials previously deemed challenging to manage. One strategy involves the development of biocomposites through extrusion without external biomass, employing natural components within the processed cells of PHA-producing microorganisms. This approach circumvents polymer purification and facilitates research on component mixing and the effects of residual endotoxins on material properties. In PHBV or mcl-PHA composites produced in this manner, the difficulties in extracting the pure polymer may suggest stronger phase binding within the mixture compared to the original biomass [138,139]. Such “inseparable” systems enable the creation of biocomposites with modified barrier and degradation kinetics. The expansion of biomass sources also includes the use of hemp and flax fibers as fillers for the PHBV matrix derived from microbiological materials [76]. Processing involves cutting fibers to approximately 1 mm and preparing granules with 15% by weight of filler; molding then produces technical elements that facilitate effective stress transfer between phases. These composites integrate mechanical properties with biodegradability under specified conditions, rendering them suitable for automotive or sustainable construction applications. Additionally, new raw materials include biochar—a carbon material produced through the thermochemical conversion of biomass with limited oxygen. As an additive in degradable polyesters, biochar enhances mechanical properties and thermal stability; its porous structure can also promote biological activity pertinent to degradation. In 3D printing, such additives can influence the aesthetics and thermal conductivity of workpieces. The literature underscores the economic viability and emphasizes that new raw materials should not compete with existing biomass processing pathways [140]. Using industrial by-products such as food, agricultural, or forestry waste reduces environmental impacts throughout the product life cycle. Specialized fibers, including jute and kenaf, can impart specific biological functions; their hydrophilicity facilitates the migration of water and enzymes, thereby accelerating decomposition. Models incorporating PLA/PHB/cellulose illustrate how natural components affect the morphology of stamped or 3D-printed materials [141,142]. Cellulose enhances impact strength following heat treatment and unifies the crystal structure of the matrix, thereby integrating mechanical and degradation effects. The incorporation of algal biomass as a poly(lactic acid) (PLA) additive reduces sheet weight through carbon dioxide foaming and increases susceptibility to microbial colonization, as its laminarate and glucans provide energy sources for compost bacteria [15]. In packaging applications, such fillers reduce the composting time of bulky products. Future research should focus on advancing technologies for diverse biomass feedstocks. This includes optimizing fiber surface modifications, such as silanization and alkalization, aligning processing parameters to prevent ingredient degradation, and assessing the chemical compatibility between bio-fillers and the polymer matrix [76,77]. Evaluating the impact of these additives on the product life cycle is essential, particularly concerning the maintenance of mechanical properties during use and the biodegradation rates post-service. The use of new feedstocks necessitates the integration of their chemical and physical properties with polymer matrix design to maintain technical parameters until degradation commences. Achieving a predictable balance between durability and biodegradability is crucial for industrial implementation and circular economy strategies [108]. Priority should be given to feedstocks for which variability and pretreatment burdens are quantified in conjunction with service performance and end-of-life conversion, rather than focusing solely on novelty.

### 8.2. Innovative Production Methods

In evaluating the potential of novel biomass raw materials as detailed in Section 8.1, it is crucial to develop production methodologies that enable their application in composites and degradable plastics with consistent performance. These technologies must consider the thermal and mechanical constraints of biodegradable polymers and the properties of new fillers, such as hygroscopicity, susceptibility to thermal degradation, or biological activity, which may affect processing conditions. A notable trend involves the integration of processing systems, where the synthesis or modification of the polymer matrix occurs simultaneously with the formation of the final product. For example, reactive extrusion of PLA with compatibilizers or polymer chain elongating agents increases molar mass and melt viscosity without a separate synthesis phase [91], thereby reducing logistical operations and production cycle costs. In composites reinforced with lignocellulosic fibers, multi-stage dosing can introduce part of the filler in the plasticizing section and the remainder in the cooling zone, minimizing fiber degradation during intensive mixing [78] and enhancing interfacial adhesion and stress distribution. Another approach involves foaming polymers by incorporating supercritical gases (e.g., CO_2_, N_2_) into extrusion or compression molding. This combination of density reduction with controlled pore structure results in shock-absorbing materials with thermal and acoustic insulation properties [91]. The inclusion of algal biomass as an active foaming agent further enhances biodegradability, as polysaccharides are readily accessible to microorganisms [15]. Precise control of matrix gas saturation and decompression is essential, as minor pressure variations can affect porosity homogeneity and mechanical properties. Advanced additive techniques, such as FFF 3D printing with composite filaments, represent another adaptation for degradable plastics. The preparation of PLA/PHA or PLA/natural fiber filaments requires control over diameter, flow stability, and moisture content [22]. Multi-material printing enables the creation of products with varying degradation times across zones, such as medical scaffolds with a slower-degrading central zone to support early tissue integration [82,83]. Progress in this area necessitates temperature-rheological profiles for each material combination to prevent defects arising from disparate processing properties. Hybrid molding combines processes within a single line [22]. This integration facilitates the creation of lightweight structures through high-pressure consolidation, simultaneously reducing weight by incorporating porosity. Innovative techniques include the biosynthesis of polyhydroxyalkanoates without the need for biomass purification, resulting in a product where the organic fraction serves as a bioactive filler [138]. The production of biocomposites requires the modification of fermentation equipment and phase separation strategies to preserve both the biofunctional component and the mechanical properties of the matrix. The resulting material meets the demands for packaging and agricultural applications, where its bioactivity can accelerate waste mineralization. Further research is directed towards the digital control of process parameters. By monitoring viscosity, temperature, and filler distribution within the mold, dynamic adjustments can be implemented to improve internal homogeneity. For PLA/PHBV composites used in automotive or electronic equipment, this approach ensures component durability before environmental degradation [71]. Overall, these innovative production methods integrate synthesis, processing, and property modification into an optimized process. The safe processing of biodegradable polymer feedstocks requires the protection of chemical structures while achieving functional properties, avoiding premature degradation during production [7], and designing morphology to influence biodegradation rates in accordance with environmental standards [28]. This model employs novel raw materials to maintain the competitiveness of biodegradable composites relative to traditional plastics. Therefore, the evaluation of process integration should focus on the preservation of material properties and the verification of disposal performance, rather than solely on throughput or reducing manufacturing steps.

### 8.3. Controlled Degradation Design

In designing controlled degradation for biodegradable plastics, it is essential to determine the chemical structure, morphology, and composition to ensure that the timing and mode of decomposition meet application requirements. While material design influences susceptibility, environmental exposure remains the primary determinant of degradation timing; it does not inherently establish an automatic end-of-service threshold. A key strategy involves managing polymer crystallinity: increased crystallinity reduces water and enzyme permeability, thereby slowing the hydrolysis of ester bonds in PLA or PHBV [28], while more amorphous phases accelerate this process. Stereochemical control, such as adjusting the L- and D-lactide ratios in PLA synthesis, affects the onset of degradation and mechanical stability during use [28].

Fillers and plasticizing or compatibilizing additives also significantly influence decomposition kinetics. Highly hydrophilic natural fibers, including flax, hemp, and bamboo, enhance moisture penetration and accelerate biodegradation [78], whereas mineral additives like ZnO or nanosilicate act as diffusion barriers [62]. Natural plasticizers, such as acetyltributyl citrate (ATBC), increase PLA chain mobility, thereby facilitating biofragmentation [15], which is beneficial in packaging applications requiring short industrial-composting times.

Multiphase composite structures are essential for engineering materials with specific degradation profiles. By integrating polymers with different degradation rates, a sequential reduction in mechanical properties can be achieved. In PLA/PHA blends, the more rapidly degrading PHA phase induces porosity, facilitating the degradation of PLA [87,88]. This feature is beneficial for medical scaffolds, where a durable core provides prolonged support, while an outer layer degrades earlier to initiate tissue regeneration. Processing conditions, including extrusion and forming parameters, are crucial because they affect the molar mass. Residual moisture and excessive thermal exposure during processing may lead to PLA chain scission, thereby reducing the molar mass prior to application. In multilayer laminates, the strategic combination of layers with distinct environmental resistances governs decomposition rates. Surface modifications of reinforcing fibers, such as silanization and alkalization, can decrease hydrophilicity and delay the onset of biodegradation [37,38,78]. Designing materials with controlled degradation requires consideration of the intended use environment. For marine applications, materials must withstand lower temperatures and different enzymatic activities compared to composting conditions; modifying PHA monomer composition can adjust decomposition rates to suit these conditions [30]. In medical applications, implant resorption must align with the healing process. Scaffold microporosity is engineered to facilitate cell colonization post-implantation while maintaining mechanical load capacity throughout the therapeutic period until structural integrity diminishes [81]. The integration of sensors or conductive nanocomponents allows for real-time monitoring of material condition, signaling impending degradation [101]. This approach aligns structural control with product life cycle management, supporting circular economy principles. Future research emphasizes the digital modeling of degradation kinetics based on processing parameters and material composition. Such simulations will enable performance predictions across various scenarios, allowing for design refinements prior to industrial production. Hybrid modification systems, which combine matrix morphology alterations (such as crystallinity control) with selectively degradable biologically active fillers, are also being explored. Distinguishing the useful service life from the end-of-life conversion constitutes a design objective rather than a definitive assurance of accurately timed decomposition [1]. These strategies seek to harmonize durability with the intended biodegradation of plastics employed in packaging, medical structures, and technical components across various industries. Table 4 provides a summary of the paired tests required to assess this objective, emphasizing that an enhancement in a single property does not inherently indicate an environmental benefit.

## 9. Conclusions

The evidence indicates that a conditional, application-specific evaluation is necessary: degradable plastics are beneficial when their functional service and end-of-life pathways are designed in tandem. While enhancements in strength, barrier performance, or processability reveal technical potential, they do not inherently establish environmental superiority. Packaging must ensure product protection and align with a compatible collection and treatment route; medical devices require functional retention followed by confirmed bioresorption; automotive components necessitate durability and feasible recovery. Matrix degradation should not be conflated with the disposal of persistent reinforcement. Consequently, no polymer family or filler can be deemed universally preferable without considering product geometry and treatment conditions [27].

The uncertainties identified here should be prioritized based on their potential to invalidate environmental claims. The primary concern is the discrepancy between laboratory disintegration and complete product mineralization under realistic treatment or release conditions (Section 5.6, Section 7.3, Section 7.4 and Section 7.5) [37,63,126]. The second concern involves the exposure-dependent fate and toxicity of oligomers, particles, and additives: the detection of PLA oligomers confirms their presence [135], whereas the adverse effects of concentrated PCL oligomers do not establish field risk for PLA or PHA [136]. The third concern is implementation: attitudes related to labeling do not confirm actual disposal behavior [137], and modeled production savings rely on collection and economic assumptions (Section 7.4) [3]. This ranking emphasizes the verification of environmental claims rather than a universal ranking of all application risks.

Research should initially pair service-life testing with polymer-specific carbon balances and residual-particle measurements on the same aged products. Subsequently, it should connect intermediate identification to measured exposure and chronic ecotoxicity, and evaluate specific disposal instructions through observed sorting and littering outcomes under local infrastructure conditions. Comparative life-cycle and cost analyses should employ equivalent function and transparent treatment scenarios. Further optimization of biomass fillers, processing, and multifunctional additives should occur within these verified limits rather than as substitutes. The central requirement is demonstrated compatibility between useful performance and a feasible, evidence-based end-of-life outcome.

## Figures and Tables

**Table 2:** Comparison of major international compostability standards for biodegradable plastics.

Standard	Scope of Application	Biodegradation	Disintegration	Typical Test Conditions	Additional Requirements
EN 13432 [39] (EU)	Packaging and packaging materials intended for recovery through composting and biodegradation	≥90% biodegradation within ≤6 months	≥90% disintegration; After 12 weeks, ≤10% of the initial dry matter can remain as a fraction of >2 mm	Controlled composting conditions; in biodegradation studies typically approx. 58 ± 2 °C	No negative impact on the process and quality of the compost; Reducing the content of metals and other components; Ecotoxicity assessment
ASTM D6400 [125] (USA)	Plastics and plastic products intended for aerobic composting in municipal and industrial installations	In newer releases, ≥90% conversion of organic carbon to CO_2_ in ≤180 days	After 84 days, ≤10% of the initial dry matter may remain on a 2 mm sieve	Aerobic and thermophilic conditions; biodegradation, e.g., according to ASTM D5338 or ISO 14855	Ingredient control and no adverse effect on compost quality and plant growth
ISO 17088 [132] (International)	Plastics and plastic products intended for organic recycling in industrial installations	≥90% biodegradation, expressed by converting organic carbon to CO_2_, in ≤180 days	After 84 days, ≤10% of the initial dry matter may remain on a 2 mm sieve	Controlled industrial composting; preferential biodegradation test at approx. 58 ± 2 °C	Plant growth and earthworm toxicity studies; control of metals, PFCs and other hazardous substances

**Table 3:** Evidence status for intermediates, microplastics, and biodegradability-related consumer responses.

Issue	Reported in the Cited Studies	Contested Inference	Unresolved Requirement
Degradation intermediates	PLA oligomers were quantified in both laboratory and agricultural soils [135]. In contrast, PCL oligomers, when present at artificially elevated concentrations in laboratory settings, elicited adverse responses [136].	Environmental safety or harm cannot be determined solely by assessing either the biocompatibility of parent polymers or the detection of oligomers. Results obtained from PCL cannot be directly applied to PLA/PHA.	Establish correlations between the concentrations and persistence of oligomers/additive mixtures and their chronic effects under realistic exposure conditions, considering relevant formulations [135,136].
Microplastic formation	PLA/PBS-based films produced microplastics upon exposure to water, contingent on specific conditions [63]. The release of fragments does not serve as an indicator of mineralization.	Rapid fragmentation does not necessarily diminish exposure, as generation and biological removal are distinct processes. The compostability of materials cannot be universally applied to all receiving environments [126].	Measure the residual particle mass, size distribution, and polymer-carbon conversion during realistic treatment periods and subsequent exposure [63].
Product labeling	A survey conducted across four countries revealed that the acceptance of leaving biodegradable packaging in natural environments is context-dependent [137]. The study assessed attitudes rather than actual littering behavior.	The assertion that labeling invariably leads to increased littering lacks empirical support. It is essential to distinguish between material beliefs, cultural context, and actual behavior [137].	Examine the causal effects of specific labels on observed sorting and littering behaviors, including the availability of the instructed treatment route in the local context [137].

**Table 4:** Coupled processing–structure–performance–degradation assessment. Qualitative design implications, not a numerical ranking.

Design Choice	Structural Consequence	Service Trade-Off	End-of-Life Test Needed
Cellulose versus woodflour in PHBV [37]	The interaction at the interface and the presence of lignin influence the accessibility of water and microorganisms.	Thermal stability and rheology change with filler type.	Assess both the disintegration and the conversion of polymer-carbon, ensuring a clear distinction between the loss of filler and the conversion of the matrix.
ATBC-containing PHBV formulations [28]	Plasticization changes chain mobility; nucleating fillers also alter morphology.	Processability and flexibility must be balanced against storage stability.	Mineralization should be compared within the intended treatment environment, rather than assessing mass-loss rates from a different environment.
Repeated melt processing of PHBV/filler composites [131]	Dispersion and matrix–filler contact change over successive cycles.	Improved homogeneity may coexist with initial mechanical losses.	Retained service properties should be paired with end-of-life testing of the reprocessed product, as recycling does not equate to mineralization.
PLA/PCL blending [126]	Changing composition creates a formulation with different degradation behavior.	Properties and geometry must remain suitable for the application.	Test the complete blend in its intended environment; do not infer its behavior from either neat polymer.

## Data Availability

No new data were created or analyzed in this study. Data sharing is not applicable to this article.

## Supplementary Materials

The following supporting information can be downloaded at: https://www.mdpi.com/article/10.3390/ma19194230/s1, Supplementary Material S1: Reported literature-search details.
