# Supplementary material for: Degradable Plastics and Their Composites: A Comprehensive Review of Production, Properties, and Applications
Source: Materials (Basel). 2026 Oct 5;19(19):4230. doi: 10.3390/ma19194230 (PMC13666331; doi:10.3390/ma19194230)
Supplement: Supplementary file 1 [file materials-19-04230-s001.zip › materials-4553628-supplementary.pdf]

materials

MDPI

Review

Degradable Plastics and Their Composites: A Comprehensive Review of Production, Properties, and Applications

Patrycja Bazan [1], Paulina Majewska [2], Kamil Badura [2,3] and Stanisław Kuciel [1,*]

[1] Faculty of Material Engineering and Physics, Cracow University of Technology, Aleja Jana Pawła II 37, 31-864 Krakow, Poland; patrycja.bazan@pk.edu.pl
[2] CUT Cracow University of Technology Doctoral School, Faculty of Material Engineering and Physics, Cracow University of Technology,
Warszawska 24, 31-155 Krakow, Poland; paulina.majewska@doktorant.pk.edu.pl (P.M.); kamil.badura@doktorant.pk.edu.pl (K.B.)
[3] SHM System Sp. z o.o., Sp. kom., Jana Pawła II St. 82A, 30-444 Libertów, Poland
* Correspondence: stanislaw.kuciel@pk.edu.pl

Supplementary Material S1

This supplement contains the detailed literature-search account reported for the review. The main text provides the condensed description.

S1.1. Databases and scope

The literature search was executed utilizing three primary sources of scientific information: Google Scholar, Web of Science, and Scopus. The search strategy was meticulously crafted to encompass fundamental aspects of degradable and biodegradable polymer materials, including their production, processing, properties, degradation mechanisms, and industrial applications.

S1.2. Search terms and combinations

The search employed both individual keywords and their combinations. Among the phrases utilized were: “degradable polymers,” “biodegradable polymers,” “biodegradable plastics,” “compostable plastics,” “bio-based polymers,” “PLA biodegradation,” “PHA biodegradation,” “PHB degradation,” “PHBV composites,” “PLA composites,” “PLA/PHA blends,” “biodegradable polymer composites,” “natural fiber reinforced biodegradable composites,” “biodegradable polymer processing,” “PLA extrusion,” “PLA injection molding,” “additive manufacturing of biodegradable polymers,” “biodegradable polymers mechanical properties,” “thermal properties of biodegradable polymers,” “barrier properties of biodegradable polymers,” “polymer degradation mechanisms,” “industrial composting biodegradable polymers,” “biodegradable plastics recycling,” “chemical recycling of PLA,” “biodegradable polymers environmental impact,” “biodegradable polymers life cycle,” “biodegradable plastics packaging,” “biodegradable polymers biomedical applications,” and “biodegradable polymer automotive applications.” Depending on the specific issue under analysis, more detailed combinations were also employed, such as “PLA and PHBV and mechanical properties,” “PHA and natural fibers and biodegradation,” “PLA and industrial composting and EN 13432,” and “biodegradable polymers, recycling and circular economy.”

Academic Editor: Philippe Evon

Received: 25 August 2026
Revised: 27 September 2026
Accepted: 02 October 2026
Published: 5 October 2026

Copyright: © 2026 by the author. Licensee MDPI, Basel, Switzerland. This article is an open access article distributed under the terms and conditions of the Creative Commons Attribution (CC BY) license.

S1.3. Screening and selection

In total, approximately 140 scientific publications were evaluated during the search and initial screening phases. Initially, the alignment of the title and abstract with the thematic scope of the study was assessed. Publications deemed potentially relevant were subsequently analyzed in full text. Peer-reviewed articles, experimental studies, and review papers on PLA, PHA, PHB, PHBV, and other degradable polyesters, including their composites and mixtures, processing technologies, and mechanical, thermal, barrier, optical, and rheological properties, as well as biodegradability, compostability, recycling, and industrial applications, were selected for further analysis.

Publications that did not directly pertain to the subject matter of the review, those with insufficient descriptions of materials or methodologies, and those duplicating information available in newer or more comprehensive studies were excluded. Preference was given to current peer-reviewed publications, although older sources were considered if they were fundamental to understanding the mechanisms of degradation, synthesis, processing, or standardization of polymeric materials. The final selection of literature was based on its alignment with the study's scope, scientific quality, and suitability for comparative evaluation of the polymer systems discussed.

https://doi.org/10.3390/ma19194230
